# A retrospective cohort study of a community-based primary care program’s effects on pharmacotherapy quality in low-income Peruvians with type 2 diabetes and hypertension

**DOI:** 10.1371/journal.pgph.0003512

**Published:** 2024-08-22

**Authors:** John E. Deaver, Gabriela M. Uchuya, Wayne R. Cohen, Janet A. Foote

**Affiliations:** 1 Asociación Siempre Salud, Chincha Alta, Ica, Peru; 2 College of Medicine, University of Arizona, Tucson, Arizona, United States of America; 3 College of Public Health, University of Arizona, Tucson, Arizona, United States of America; Sun Yat-Sen University, CHINA

## Abstract

Little is known about the effects of the Chronic Care Model (CCM) and community health workers (CHWs) on pharmacotherapy of type 2 diabetes and hypertension in resource-poor settings. This retrospective cohort implementation study evaluated the effects of a community-based program consisting of CCM, CHWs, guidelines-based treatment protocols, and inexpensive freely accessible medications on type 2 diabetes and hypertension pharmacotherapy quality. A door-to-door household survey identified 856 adults 35 years of age and older living in a low-income Peruvian community, of whom 83% participated in screening for diabetes and hypertension. Patients with confirmed type 2 diabetes and/or hypertension participated in the program’s weekly to monthly visits for < = 27 months. The program was implemented as two care periods employed sequentially. During home care, CHWs made weekly home visits and a physician made treatment decisions remotely. During subsequent clinic care, a physician attended patients in a centralized clinic. The study compared the effects of program (pre- versus post-) (N = 262 observations), and home versus clinic care periods (N = 211 observations) on standards of treatment with hypoglycemic and antihypertensive agents, angiotensin converting enzyme inhibitors, and low-dose aspirin. During the program, 80% and 50% achieved hypoglycemic and antihypertensive standards, respectively, compared to 35% and 8% prior to the program, RRs 2.29 (1.72–3.04, p <0.001) and 6.64 (3.17–13.9, p<0.001). Achievement of treatment standards was not improved by clinic compared to home care (RRs 1.0 +/- 0.08). In both care periods, longer retention in care (>50% of allowable time) was associated with achievement of all treatment standards. 85% compared to 56% achieved the hypoglycemic treatment standard with longer and shorter retention, respectively, RR 1.52 (1.13–2.06, p<0.001); 56% compared to 27% achieved the antihypertensive standard, RR 2.11 (1.29–3.45, p<0.001). In a dose-dependent manner, the community-based program was associated with improved guidelines-based pharmacotherapy of type 2 diabetes and hypertension.

## Introduction

Cardiovascular disease (CVD), which accounts for the most life years lost due to premature adult mortality, is in most cases attributable to modifiable risk factors including hypertension and diabetes [1]. Hypertension and type 2 diabetes are frequently comorbid, and their treatment options overlap.

This study focuses on pharmacotherapy of hypertension and type 2 diabetes in a low-income urban community in Peru. Blood pressure (BP) and blood glucose are risk factors (or intermediate clinical outcomes) because of their association with complications. Blood pressure (BP) reduction prevents macrovascular (cardiovascular) complications of hypertension [2]. Blood pressure (BP) [3] and blood glucose (BG) [4] reduction prevent both microvascular and cardiovascular complications of diabetes [3, 4]. Most patients are maximally protected from CVD when they achieve target levels (thresholds) of hemoglobin A1c (a measure of long-term glycemia) and BP.

Globally, approximately 10% [5] and 30% [6] of adults are affected by diabetes or hypertension, respectively. Both are increasing in prevalence, disproportionately so in low and middle-income countries (LMICs) [7, 8]. Primary health care is poorly adapted to chronic disease management in many LMICs, which also experience shortages of trained workers and essential medications and technologies [9]. Several models of care have been proposed to address these issues. The Chronic Care Model (CCM) [10] and Innovative Care for Chronic Conditions (ICCC) [11] frameworks envision care of chronic diseases that is patient-centered, team-based, uninterrupted, proactive, planned, and preventive. The American Diabetes Association, in its guidelines, regards CCM as an “effective framework for improving the quality of diabetes care” [12].

Risk factor control is poor in most countries. Among patients with type 2 diabetes and hypertension in the USA, only about half attain glycemic [13] and BP control [14], and in LMICs only 23% and 10% attain glycemic [15] and blood pressure [16] control, respectively. The difference between countries is attributable, in part, to differences in screening and diagnosis. In the USA, 74–79% of persons with diabetes or hypertension are diagnosis-aware [13, 14], compared to 39–44% in LMICs [15, 16].

There is evidence of beneficial effects of CCM and CHWs on risk factor control in patients with diabetes and hypertension. Multiple components of CCM have positive effects on hemoglobin A1c (HbA1c) or fasting glucose reduction or glycemic control [17, 18], and reduced or controlled blood pressure [17], in patients with diabetes in high-income countries (HICs) [17] and LMICs [18]. Systematic reviews have also found evidence for positive effects of CHWs, especially when delivering self-management education and support, on reduced or controlled blood pressure in patients with hypertension, in both HICs [19, 20] and LMICs [21]; and on HbA1c reduction or glycemic control in patients with diabetes, in HICs [22]. Multicomponent interventions utilize multiple elements of CCM [23] or similar health system-strengthening measures [18]. Two or more elements have greater effects than any single intervention on BP control [18] and four or more CCM elements had greater effects on glycemic control (compared to fewer) [17].

Treatment intensification is defined as initiation of, or switch to, a new medication or increased dosage of existing medication when indicated by glycemic measures or BP that exceed targets [24–28]. Clinical inertia (the failure of appropriate treatment intensification) is common in the care of type 2 diabetes [28] and hypertension [29] and is the most important physician-level contributor to poor glycemic control [30].

Risk factors (intermediate clinical outcomes) like glycemic measures and BP are commonly used to measure quality. They are generally considered as thresholds. However, risk does not have a dichotomous relation to risk factors. Values of relative risk reduction per increment of decline in risk factors, e.g., HbA1c [4] and BP [3], in diabetes, and SBP in hypertension [2], are constants across a wide range of absolute risk. Therefore, absolute risk reduction, for the same increment of decline, is greater when pre-treatment risk is higher. Threshold measures do not account for this benefit, which occurs even if targets are not achieved [31]. Poor control can occur despite treatment intensification or because of clinical inertia, wherein glycemic measures and BP might be improved in the former (albeit above threshold) and unimproved in the latter. Threshold measures identify poorly controlled patients without making this distinction. However, clinical action measures [24, 32], also known as “tightly linked” [25, 26] or “pathway” measures [27] or “sequential indicators” [28] make this distinction and, further, they measure the velocity of intensification. They link clinical criteria, e.g., BP, and treatments that occur at separate times. (Hereafter, we use the term “clinical action measure” to encompass all such longitudinal measures). For example, high-quality care is indicated by a controlled risk factor or, if uncontrolled, then treatment intensification within a specified time. Time to intensification is an independent risk factor for microvascular [33] and cardiovascular [34] complications of type 2 diabetes. Early treatment intensification reduces glycemic burden, thereby preserving pancreatic beta-cell function, resulting in earlier and more durable glycemic control using less medication [35]. Treatment intensification, unlike other physician-level process measures (numbers of tests and exams), has beneficial effects on clinical outcomes [36], including long-term complications [34]. Our community-based program employed four components of CCM and included cost-free access to inexpensive generic medications and a period of CHW home visits with remote physician treatment decisions. Our primary aim was to evaluate the effects of the overall program on four guideline-defined treatment standards constructed as “clinical action measures”. These were 1) hypoglycemic treatment, 2) antihypertensive treatment, 3) use of ACEi in type 2 diabetes, and 4) low-dose aspirin for primary CVD prevention. Secondary aims were evaluate the effects of care periods embedded within the program (clinic versus home care), and retention in care period (longer versus shorter duration) on treatment standards achievement.

## Methods

### Study design and setting

Asociación Siempre Salud is a Peruvian nonprofit organization that works to prevent premature adult mortality in the Chincha District of Peru. Beginning in 2008 in the aftermath of an earthquake, the organization deployed temporary “popup” clinics, approximately every two months, in schools, churches, and tents. In 2011, Siempre Salud began operation of the primary care program which we describe in this report.

This was a retrospective cohort implementation study that compared effects on the fidelity of treatment standards (process variables). Post-program observations were compared to pre-program observations (historical controls) and care period observations (clinic versus home care) were also compared. De-identified patient data for the study were extracted from extant data that had been routinely collected during Siempre Salud’s operation of a community-based type 2 diabetes and hypertension care program that lasted 27 months and ended in 2014. There was no patient follow-up thereafter. We complied with all applicable items in Strengthening the Reporting of Observational Studies in Epidemiology (STROBE) checklist for cohort studies [37] (S2 File). This observational study is registered at ClinicalTrials.gov (identifier NCT05979142) [38].

The community-based program was conducted in three neighborhoods (“the community”) in Pueblo Nuevo, Chincha District, Peru. Most people in the study community live below the poverty line [39]. Door-to-door surveys identified all persons residing in the community. Persons > 35 years old were eligible, without exclusions, to participate in type 2 diabetes and hypertension screening and diagnosis. Most participated in mass screenings between September and December 2011, others in later ad hoc screenings. Patients on medications with negative screening results were re-screened following medication withdrawal. Those with confirmed diagnoses were eligible to participate in the program.

### Data collection

During the community-based program, Asociación Siempre Salud entered data from structured encounter forms into a spreadsheet (Microsoft Excel, Microsoft Corporation, Redmond, WA, USA) during normal program operation for the purpose of auditing quality of care. The de-identified data was shared with the University of Arizona College of Public Health according to the terms of a Data Use Agreement for Limited Data Sets. This de-identified data was accessed for research purposes beginning on January 23, 2020. All de-identified individual participant data extracted during the study is available from the Dryad repository [40] (with no end date) to anyone who wishes to access the data for any purpose.

### Ethics statement

The University of Arizona Institutional Review Board determined that human subjects review was not required for this study (Protocol Number 1912252903). The study did not obtain consent from patients for use of their de-identified data in this study. All data had been gathered prior to study onset. During the study, no authors had access to data that could identify individual study participants.

### Exposures

Three binary exposures were evaluated for their effects on achievement of four treatment standards and one composite standard: 1) ‘program exposure’ (post- vs. pre-exposure); 2) ‘care period’ (clinic compared to home care), and 3) ‘retention in care period’ (>50% of allowable time versus less).

### Program exposure

The Peruvian Ministry of Health provides care to 70% of Peru’s population including uninsured patients and others covered by the public insurance program, Seguro Integral de Salud (SIS) [41]. During the pre-exposure period, usual care was provided by several Ministry of Health clinics, two public hospitals, private clinics and pharmacies, and Siempre Salud (the latter as described in ’Study design and setting’).

The program, to which patients were then exposed, had components of four CCM elements (delivery system design, self-management, decision support, and community resources), and care and medications that were cost-free to patients. See S1 File for a full program description. The program was delivered using two care models employed sequentially: first home care (CHW home visits and remote physician treatment decisions), then clinic care (see ‘care period’ exposure below). Self-management was based on the American Association of Diabetic Educators (AADE) national standards [42] and utilized patient educational materials for diabetes and hypertension obtained from professional societies and government agencies in the USA. Decision support consisted of guidelines-based standards and the treatment protocols (S1 and S2 Tables respectively). Standards were created by reconciling guidelines for care of diabetes [43–48], hypertension [47–49] and primary prevention of CVD [48, 50] and adopting those that were suitable to our low-resource setting. Laboratory tests (other than blood glucose concentration, and external laboratory testing of serum potassium and creatinine) were generally unavailable. Specialist physicians, diabetes educators, and registered dieticians were not accessible and two important medications relevant to diabetes and hypertension management (insulin and a statin drug) were too costly for the program. Condensed treatment protocols were created that used seven medications: two classes of hypoglycemic agents (metformin and a sulfonylurea); four antihypertensive drug classes (angiotensin converting enzyme inhibitor (ACEi) or angiotensin receptor blocker (ARB), thiazide diuretic, calcium-channel blocker (CCB), and beta-receptor blocker (BB)); and low-dose aspirin. The standards and medication protocols are found in S1 and S2 Tables, respectively.

### Care period exposure

During the ‘home’ care period, the physician made an initial visit, after which CHWs made weekly visits to patients’ homes. CHWs monitored clinical parameters, provided self-management education and support, tracked self-care behaviors, documented visits, acted on clinical alerts, and delivered medications. They entered encounter data into a spreadsheet and filled prescriptions under physician supervision. The physician made treatment decisions remotely based on home visit data reviewed during monthly treatment decision conferences attended only by the physician and CHWs. Occasionally treatment decisions required in-person visits, in which cases the physician saw patients in their homes. Prescriptions were dispensed directly by Siempre Salud. There were no pharmacy visits. Patients remained at home for the entirety of their care except for occasional laboratory visits. A six-month hiatus (an inability to finance operations) occurred before the subsequent ‘clinic’ care period. During the ‘clinic’ period, patients made monthly visits to a centralized clinic, where the same physician provided all care; there were no home visits; and CHWs functioned as clinic assistants. They conducted intake and filled prescriptions under physician supervision.

### Retention in care period exposure

Greater than 50% retention in care period, i.e., 9 or more (of 17 allowable) months in the clinic or 6 or more (of 10 allowable) months in home care; was compared to < = 50% retention corresponding to 8 or fewer months in the clinic, or 5 or fewer months in home care.

### Outcomes measures

In this study, four binary clinical action measures (treatment standards) and a composite measure were coded “yes” (achieved) or “no” (not achieved). Other binary variables were coded “yes’ or “no” unless stated otherwise. Medications and doses were obtained by patient self-reports or, once enrolled in the program, from home and clinic encounter records. Maximum doses of hypoglycemic or antihypertensive agents, low-dose aspirin, and ACEi were coded ‘yes’ if ‘ever received’ during the program, regardless of when or for how long.

The treatment protocols (S2 Table) for type 2 diabetes and hypertension required medication titration in steps made every two to four weeks (repeated treatment intensification). Titration (intensification) stopped at one of two points: 1) glycemic or blood pressure control, or 2) the end of the pathway (in the absence of control).

1. The hypoglycemic treatment standard for type 2 diabetes was, therefore, defined as glycemic control or the end of the treatment pathway, i.e., maximum doses of two oral hypoglycemic agents. Type 2 diabetes was diagnosed using ADA criteria [43] of fasting capillary blood glucose values 7.0 mmol/L or greater on two occasions (or, in one case, a random glucose >11 mmol/L) measured with Hemocue Glucose 201 System (Hemocue AB, Angelholm, Sweden). Glycemic control was defined as fasting glucose <8.7 mmol/L (ADA standard <7.2 mmol/L [43] +20%). Fasting glucose was the average value obtained during screening and diagnosis (pre-exposure) or the median of monthly average values obtained with NovaMax glucometers (Nova Biomedical, Inc., Waltham, MA, USA) during the program or care periods (post-exposure).
2. The antihypertensive treatment standard was defined as BP control or the end of the treatment pathway, i.e., maximum of three or more antihypertensive agents. All patients with elevated BP were eligible. Elevated blood pressure included hypertension, defined as systolic BP (SBP) > = 140 mm Hg or diastolic BP (DBP) > = 90 mm Hg [49] and, in patients with diabetes, SBP > = 130 mm Hg or DBP > = 80 mm Hg [43, 49]. BP was measured using an automated device (Omron Healthcare, Inc. Kyoto, Japan). Hypertension was diagnosed if the SBP was > = 140 mm Hg or DBP was > = 90 mm Hg (as the average of two BP measurements) and persisted during a second set of BP measurement taken at home at least one week later. BP control was defined as SBP <140 mm Hg and DBP<90 mm Hg in patients with hypertension only and SBP <130 and DBP <80 in patients with diabetes. BP values were the average SBP and DBP during screening and diagnosis (pre-exposure) or the median of monthly average values obtained during the program or care periods (post-exposure).
3. The ACEi in diabetes standard was defined as use of any ACEi in patients with diabetes and elevated BP (>130/80 mm Hg).
4. The low-dose aspirin standard was defined as any low-dose aspirin in patients with 10-year CVD risk >10%. CVD risk was calculated using the Framingham Heart Study alternative model that uses non-laboratory predictors [51] (equations obtained from Table 1 of that study’s online Data Supplement). The aspirin use policy was in revision during the home care period. Therefore, aspirin treatment was not evaluated in the comparisons of care periods.
5. A composite measure in all patients was coded ‘yes’ if all treatment standards, for which patient was eligible, were achieved. A composite of four standards was used in the evaluation of the ‘program’ exposure and three standards in the evaluation of ‘care period’ (and ‘retention in care period’).

**Table 1:** Pre-program observations by diagnosis history, new versus pre-existing.

Group characteristics				
Pre-program observations, N = 131	Total, N = 131 (100%)	New, N = 44 (34%)	Pre-existing, N = 87 (66%)	p-value *
Variable	N (%)	N (%)	N (%)	
Age (years)				
35–44	25 (19%)	14 (32%)	11 (13%)	0.017
45–54	36 (27%)	11 (25%)	25 (29%)	0.685
55–64	38 (29%)	9 (20%)	29 (33%)	0.155
> = 65	32 (24%)	10 (23%)	22 (25%)	0.832
Gender female	85 (65%)	27 (61%)	58 (67%)	0.566
Pre-intervention primary care source				
Pharmacies and hospital ED †	67 (51%)	41 (93%)	23 (26%)	<0.001
Ministry of Health clinic	42 (32%)	2 (5%)	40 (46%)	<0.001
Siempre Salud ‡	25 (19%	1 (2%)	24 (28%)	<0.001
Obese ¶	64 (52%)	29 (71%)	35 (42%)	0.004
10-yr CVD risk > = 10% §, ¶	101 (81%)	29 (71%)	72 (87%)	0.048
Statin candidate §, **, ††	65 (52%)	17 (41%)	48 (56%)	0.131
Elevated blood pressure (BP)	93 (71%)	31 (70%)	62 (71%)	1.000
Type 2 diabetes	101 (77%)	31 (70%)	70 (80%)	0.271
Subgroup characteristics				
Type 2 diabetes, N = 101	Total, N = 101 (100%)	New, N = 31 (31%)	Pre-existing, N = 70 (69%)	
BP > = 130/80, N = 63 including 31 with hypertension	63 (62%)	18 (58%)	45 (64%)	0.657
Elevated blood pressure, N = 93, including 61 with hypertension	Total, N = 93 (100%)	New, N = 31 (33%))	Pre-existing, N = 62 (67%)	
Hypertension alone, N = 30	30 (32%)	13 (42%)	17 (27%)	0.168
Hypertension and diabetes, N = 31	31 (33%)	8 (26%)	23 (37%)	0.353
Diabetic BP 130-139/80-89, N = 32	32 (34%)	10 (32%)	22 (35%)	0.820
Subgroup treatment				
CVD risk > = 10% by FHS, N = 101	Total, N = 101 (100%)	New, N = 29 (29%)	Pre-existing, N = 72 (71%)	
Taking low-dose aspirin	5 (5%)	0 (0%)	5 (7%)	0.318
Statin candidate FHS/ADA	Total, N = 65 (100%)	New, N = 17 (26%)	Pre-existing, N = 48 (74%)	
Taking statin	0 (0%)	0 (0%)	0 (0%)	-
Hypertension, N = 61	Total, N = 61 (100%)	New, N = 21 (34%)	Pre-existing, N = 40 (66%)	
Poor control (BP > = 140/90)	57 (93%)	21 (100%)	36 (90%)	0.289
Receiving any antihypertensive	29 (48%)	3 (14%)	26 (65%)	<0.001
Type 2 diabetes, N = 101	Total, N = 101 (100%)	New, N = 31 (31%)	Pre-existing, N = 70 (69%)	
Poor glycemic control (fbs > = 7.2 mmol/L) ‡‡	91 (91%)	30 (97%)	61 (88%)	0.267
Poor glycemic control (fbs > = 8.7 mmol/L) ‡‡	68 (68%)	19 (61%)	49 (71%)	0.361
Receiving any hypoglycemic agent	49 (49%)	0 (0%)	49 (70%)	<0.001
Type 2 diabetes BP > = 130/80, N = 63, including 31 with hypertension	Total, N = 63 (100%)	New, N = 18 (29%)	Pre-existing, N = 45 (71%)	
Poor control (BP > = 130/80)	57 (90%)	18 (100%)	39 (87%)	0.170
Receiving any antihypertensive	23 (37%)	3 (17%	20 (44%)	0.047
Receiving ACEi	19 (30%)	1 (6%)	18 (40%)	0.007

* Fisher’s exact test 2-sided p-value

† ED = emergency department

‡ Asociación Siempre Salud “pop-up” clinics 2008–2011; ¶ N = 124, 7 missing BMI

§ 10-year CVD risk using alternate Framingham Heart Study (FHS) score based on body mass index (BMI) (not cholesterol)

** statin-eligible by American Diabetes Associaion (ADA) (diabetes >40 years old with hypertension) and World Health Organization Package of Essential Noncommunicable Disease Intervention (WHO PEN) (10-year CVD risk >20%), the latter by alternate FHS risk score

†† 5 missing, 7 missing BMI, needed for CVD risk calculation, but 2 of those were eligible by the above ADA criteria

‡‡ N = 100, 1 missing pre-program fasting glucose. ACEi = angiotensin converting enzyme inhibitor.

### Covariates

‘Diagnosis history’ was coded ‘pre-existing’ if a self-reported diagnosis of diabetes or hypertension pre-dated the program; and coded ‘new’ if either diagnosis was first made following pre-program screening. Diabetes with co-existing hypertension was coded based on the diabetes history regardless of whether hypertension was new or pre-existing. All diagnoses were confirmed by diagnostic testing. ‘Sex’ was self-reported and coded ‘male’ or ‘female’. ‘Age’ was coded as a binary variable (‘<65’, ‘> = 65 years old’). ‘Obese’ was coded as ‘yes’ if body mass index (BMI) was > = 30 kg/m2 according to the WHO definition [52]. ‘Primary care source’ was coded, ‘Ministry of Health clinic’, ‘Siempre Salud clinic’, or ‘pharmacy or hospital emergency department’. Additional binary covariates, in studies of the care period exposure, were pre-program treatment with ‘any antihypertensive’ and ‘any hypoglycemic’ agent (self-reported), and pre-program ‘poor BP control’ and ‘poor glycemic control’ (based on values obtained during screening and diagnosis).

### Data analysis

The analysis of ‘program’ effects had a sample size of 262 observations i.e., two observations (pre-program and post-program) for each of 131 patients. The analysis of ‘care period’ and ‘retention in care period’ effects had a sample size of 211 observations consisting of 109 home and 102 clinic observations. Eighty patients participated in both home and clinic care and, thus, contributed two care period observations each. We omitted observations with missing data (complete case analysis).

### Statistical analysis

Statistical analyses were performed using Stata IC version 16.1 (StataCorp, College Station, Texas, USA). For each of three exposures and five outcomes, risks, relative risks, and 95% confidence intervals were calculated and two-sided p-values using Fisher’s exact test were obtained for exposure-outcome, exposure-covariate, and outcome-covariate associations. The Mantel-Haenszel test of homogeneity was used to evaluate modification of program effects by diagnosis history, and care period effects by retention in care. Covariates with both exposure and outcome associations having P-values of <0.25 were considered candidates for potential confounding. Potential confounders, effect modifiers, and independent effects were evaluated by multiple logistic regression. Full and nested regression models, differing by one variable at a time, were compared using likelihood ratio tests. Predicted probabilities were obtained by exponentiation of sums of coefficients. The 95% confidence intervals on the predicted probabilities were obtained using the method demonstrated by Inlow [53]. Durations of maximum hypoglycemic and antihypertensive treatment had non-normal distributions; Wilcoxon rank-sum tests were used for hypothesis testing of differences in medians. The motivation for the program was practical and not investigational, so there was no sample size or power calculation a priori. For findings of statistically insignificant exposure effects on the composite measure of treatment standards achievement, a post hoc power calculation was performed in Stata with the assumption of a two-sided two-sample Chi-squared test of proportions.

## Results

### Missing values

Missing values are as follows (quantity in parentheses): pre-program observations (N = 131 patients)—pre-program fasting glucose (1) and BMI (7); ‘program’ exposure (pre-post paired sample) (N = 262)—fasting glucose (2) and BMI (14); ‘care period’ exposure (N = 211)—fasting glucose (7). When BMI was missing, the Framingham CVD risk score was also missing.

### Cohort formation

**Fig 1:**
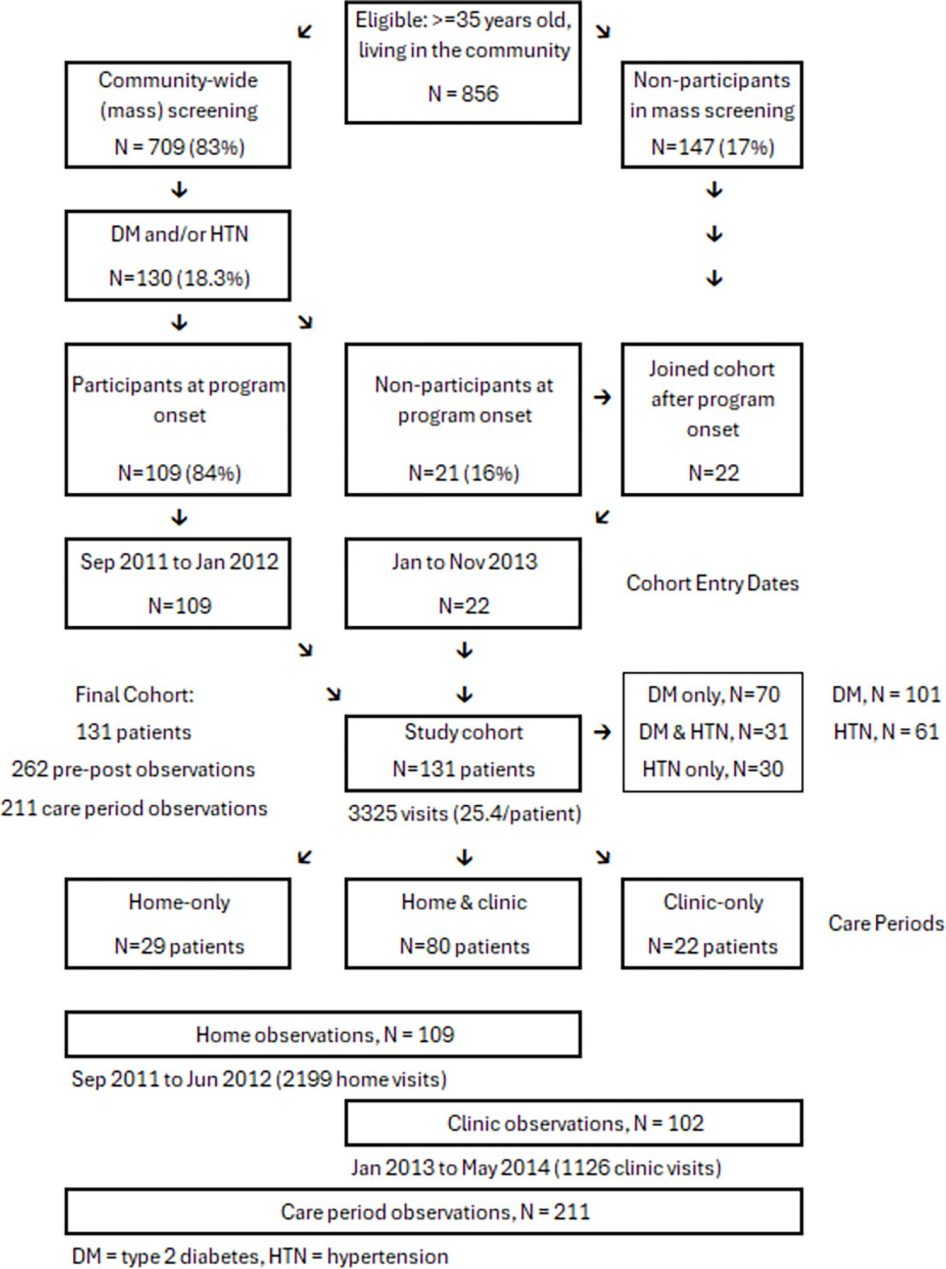
Formation of the cohort of low‐income Peruvians with type 2 diabetes (DM) and/or hypertension (HTN).

As shown in Fig 1, 83% (709/856) of eligible adults participated in mass screening. Of these, 18.3% (130/709) were diagnosed with type 2 diabetes and/or hypertension; 84% (109/130) of those participated in the program at its onset. Later, 22 patients, who had not been previously screened or who were non-participants at program onset, joined to complete the final cohort of 131 patients. The cohort produced 131 pre-post pairs (N = 262) and 211 care period observations (109 home and 102 clinic). During the program, there were 3,325 visits (2,199 home and 1,126 clinic visits).

### Baseline (pre-program) characteristics

In the 131 pre-program observations, baseline comorbid metabolic risk factors were common (Table 1). Elevated blood pressure (N = 93) includes hypertension (> = 140/90 mm Hg) (N = 61) and diabetes with blood pressure 130-139/80-89 mm Hg (N = 32). Most were obese and 62% of diabetic patients (63/101) had elevated blood pressure. Patients with new diagnoses of diabetes and/or hypertension (diagnosis-unaware at time of screening) were proportionately younger, more often obese, and had less cardiovascular risk than those with pre-existing diabetes and/or hypertension (diagnosis-aware at time of screening). Most patients with new diagnoses used pharmacies and hospital emergency departments for health care. Approximately one-third of diabetes and/or hypertension were new diagnoses and, as expected, were essentially untreated (except for three new diabetic patients taking pre-program antihypertensive agents). At baseline, approximately two-thirds of patients with pre-existing hypertension or diabetes were taking antihypertensive or hypoglycemic agents, respectively, but blood pressure and glycemic control were not significantly different comparing pre-existing and new diagnoses.

### Program effects

**Fig 2:**
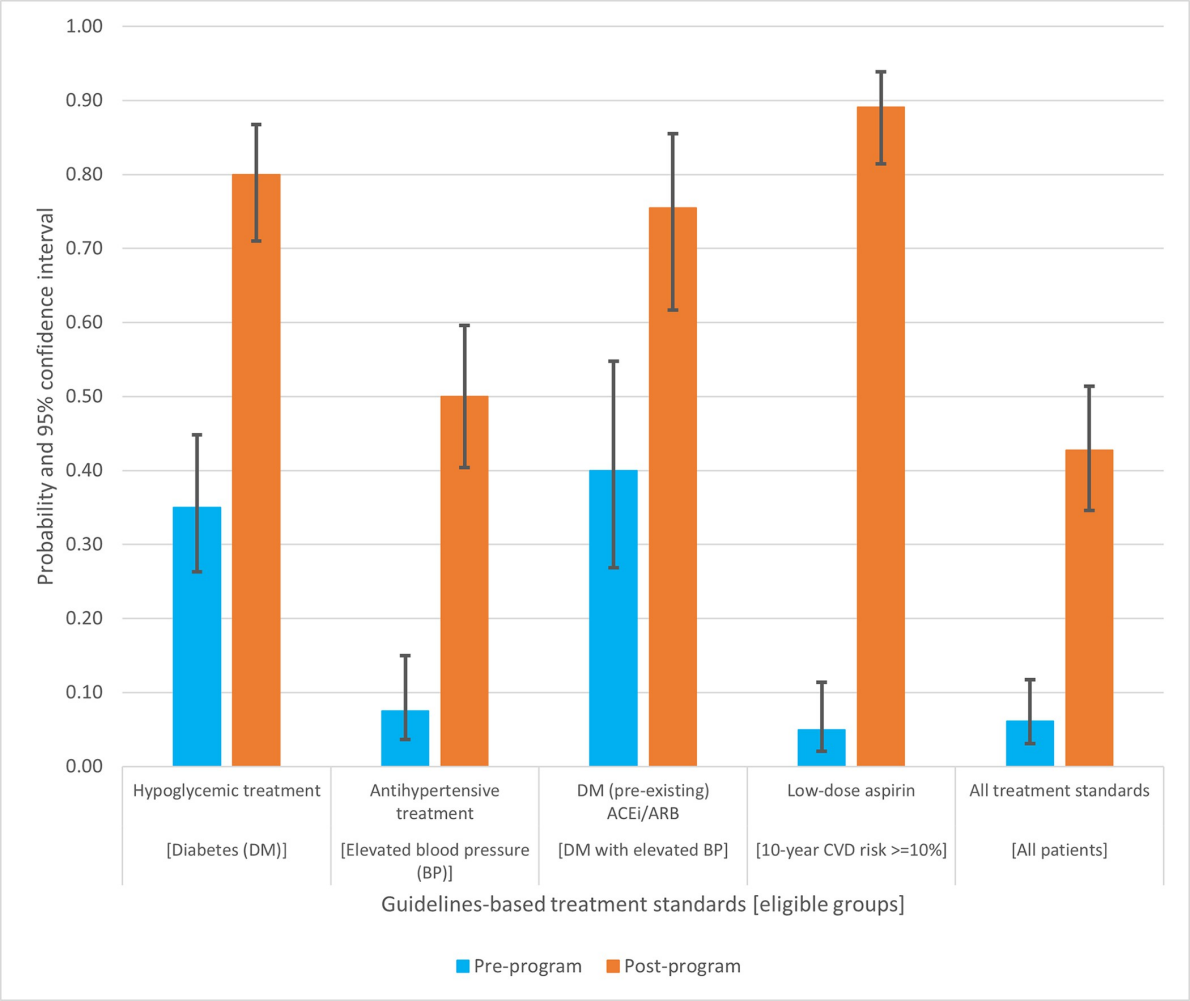
Probabilities of guidelines‐based treatment standards achievement, by program exposure (’post’ versus ’pre’).

Table 2, Part A, and Fig 2 show the program’s effects on treatment standard achievement. Across all four treatment-eligible groups, post-program standards achievement was significantly increased compared to the pre-program period, ranging from 50% to 89%, being lowest for antihypertensive treatment and highest for the low-dose aspirin standard. Forty-three percent of post-program observations achieved all standards for which they were eligible, significantly greater than 6% during the pre-program period. Confounding control in the analysis of the program exposure was provided by matching.

**Table 2:** Unadjusted effects of exposures on treatment standards achievement.

Part A. Program (post- versus pre-) effects on treatment standards achievement (’yes’ versus ’no’), N = 262								
Treatment standard	Treatment-eligible	N	R	N	R	RR	95% CI	P ††
		Post	Yes	Pre	Yes			
Hypoglycemic treatment	All DM, N = 200 *	100	0.80	100	0.35	2.29	1.72–3.04	<0.001
Controlled glycemia	All DM, N = 200 *	100	0.52	100	0.32	1.63	1.15–2.29	0.006
Antihypertensive treatment	Elevated BP, N = 195 †	102	0.50	93	0.08	6.64	3.17–13.9	<0.001
Controlled BP	Elevated BP, N = 195 †	102	0.42	93	0.06	6.53	2.92–14.6	<0.001
Diabetic ACEi/ARB	Elevated BP, DM, N = 135 ‡	72	0.76	63	0.30	2.53	1.70–3.77	<0.001
Pre-existing diagnosis	Elevated BP, DM, N = 94 ‡	49	0.76	45	0.40	1.89	1.28–2.79	0.001
Low-dose aspirin	CVD risk >10%, N = 202 ¶, §	101	0.89	101	0.05	18.00	7.64–42.4	<0.001
Composite (4 standards)	All observations, N = 262	131	0.43	131	0.06	7.00	3.48–14.1	<0.001
Part B. Care period (’clinic’ versus ’home’) effects on treatment standards achievement (’yes’ versus ’no’), N = 211								
Treatment standard	Treatment-eligible	N	R	N	R	RR	95% CI	P ††
		Clinic	Yes	Home	Yes			
Hypoglycemic	All DM, N = 153 ‡‡	78	0.81	75	0.75	1.08	0.91–1.28	0.438
Controlled glycemia	All DM, N = 153 ‡‡	78	0.50	75	0.53	0.94	0.69–1.27	0.747
Antihypertensive	All elevated BP, N = 167	77	0.49	90	0.46	1.08	0.79–1.49	0.644
Controlled BP	All elevated BP, N = 167	77	0.40	90	0.42	0.95	0.66–1.37	0.875
Diabetic ACEi/ARB	Elevated BP in DM, N = 116	55	0.60	61	0.66	0.92	0.69–1.21	0.568
Composite (3 standards)	All observations, N = 211	102	0.39	109	0.37	1.07	0.76–1.51	0.777
Part C. ’Retention in care period’ effects on treatment standards achievement (’yes’ versus ’no’). Exposed (Exp) >50% allowable time in care period. Unexposed (Unexp) < = 50% allowable time. N = 211								
Treatment standard	Treatment-eligible	N	R	N	R	RR	95% CI	P ††
		Exp	Yes	Unexp	Yes			
Hypoglycemic	All DM, N = 153 ‡‡	117	0.85	36	0.56	1.52	1.13–2.06	0.001
Antihypertensive	All elevated BP, N = 167	118	0.56	49	0.27	2.11	1.29–3.45	0.001
Diabetic ACEi/ARB	Elevated BP in DM, N = 116	86	0.70	30	0.43	1.61	1.05–2.48	0.015
Composite (3 standards)	All observations, N = 211	151	0.46	60	0.17	2.78	1.54–5.02	<0.001

DM = diabetes; BP = blood pressure; R = risk (probability); RR = relative risk, CI = confidence interval.

* missing 1 ’pre’ & 1 ’post’ fasting glucose

† hypertension (> = 140/90) or any diabetic BP > = 130/80 mm Hg

‡ diabetes with BP > = 130/> = 80 mm Hg; ¶ 10-year CVD risk using alternate Framingham score that uses clinical factors onlyBMI (not cholesterol)

§ 14 missing: 7 missing CVD risk score (no BMI) x 2 observations per patient (pre and post)

†† two-sided Fisher’s exact test p-value

‡‡ 7 missing fasting glucose.

Eighty percent of post-program observations achieved the hypoglycemic treatment standard, more than twice that during the pre-program period. Of all post-program diabetic observations, 52% had glycemic control and 28% were poorly controlled despite maximal hypoglycemic treatment.

Half of post-program observations achieved the antihypertensive treatment standard, more than six times greater than the pre-program period. Of all post-program observations with elevated blood pressure (hypertension or diabetes with BP >130/80 mm Hg), 42% of had controlled blood pressure and 8% were poorly controlled despite maximal antihypertensive treatment.

The effect of the program on ACEi treatment in diabetes (with elevated blood pressure) was modified by diagnosis history (new versus pre-existing) (M-H test p-value 0.020). About three-fourths of all eligible post-program observations with pre-existing diabetes achieved the standard, nearly twice that during the pre-program period. Otherwise, program effects on treatment standards achievement were not modified by diagnosis history.

### Care period effects

The effects of the ‘care period’ exposure on treatment standards achievement is shown in Table 2, Part B. There were no statistically significant differences between care periods, ‘home’ and ‘clinic’, in achievement of any standard. All RRs were 1.0 +/- 0.08, and there was no statistically significant difference in achievement of the composite measure. The sample size of 211 had 80% power to detect a difference in care periods of 20% in either direction, e.g., 40% versus 60%, in achievement of the composite measure. The analysis of care period effects was underpowered to detect smaller but perhaps still clinically important differences. In multiple logistic regression, none of the candidate covariates for potential confounding met criteria for model inclusion. Retention in care period acted as an independent variable and was modeled as a separate exposure.

Maximum treatment, once achieved, was durable. Fifty-nine observations had titration to maximum hypoglycemic treatment, the median duration of which was 6.6 months (IQR 3.9 to 8.3) in the shorter home period and 12.7 months (IQR 7.1 to 15.7) in the clinic period (P = 0.001). Thirteen observations had titration to maximum antihypertensive treatment, the median duration of which was 3.6 months (IQR 2.1 to 4.6) in the shorter home period, and 14.4 months (IQR 10.0 to 16.4) in the clinic period (P = 0.006). There was no association between median durations of maximum hypoglycemic or antihypertensive treatment and glycemic or blood pressure control, respectively.

### Retention in care effects

For the entire sample of 211 care period observations, 72% (151/211) was retained for more than half of allowable time, i.e., “longer”. There was no statistically significant difference between home and clinic care and retention in care as a percentage of time allowable. There was a significant association between retention in care period and achievement of each of the treatment standards including the composite measure. As shown in Table 2, Part C, longer retention had relative risks of 1.5 to almost 3 times greater achievement across all standards. Longer retention resulted in 85% and 56% achievement of hypoglycemic and antihypertensive treatment standards respectively.

**Fig 3:**
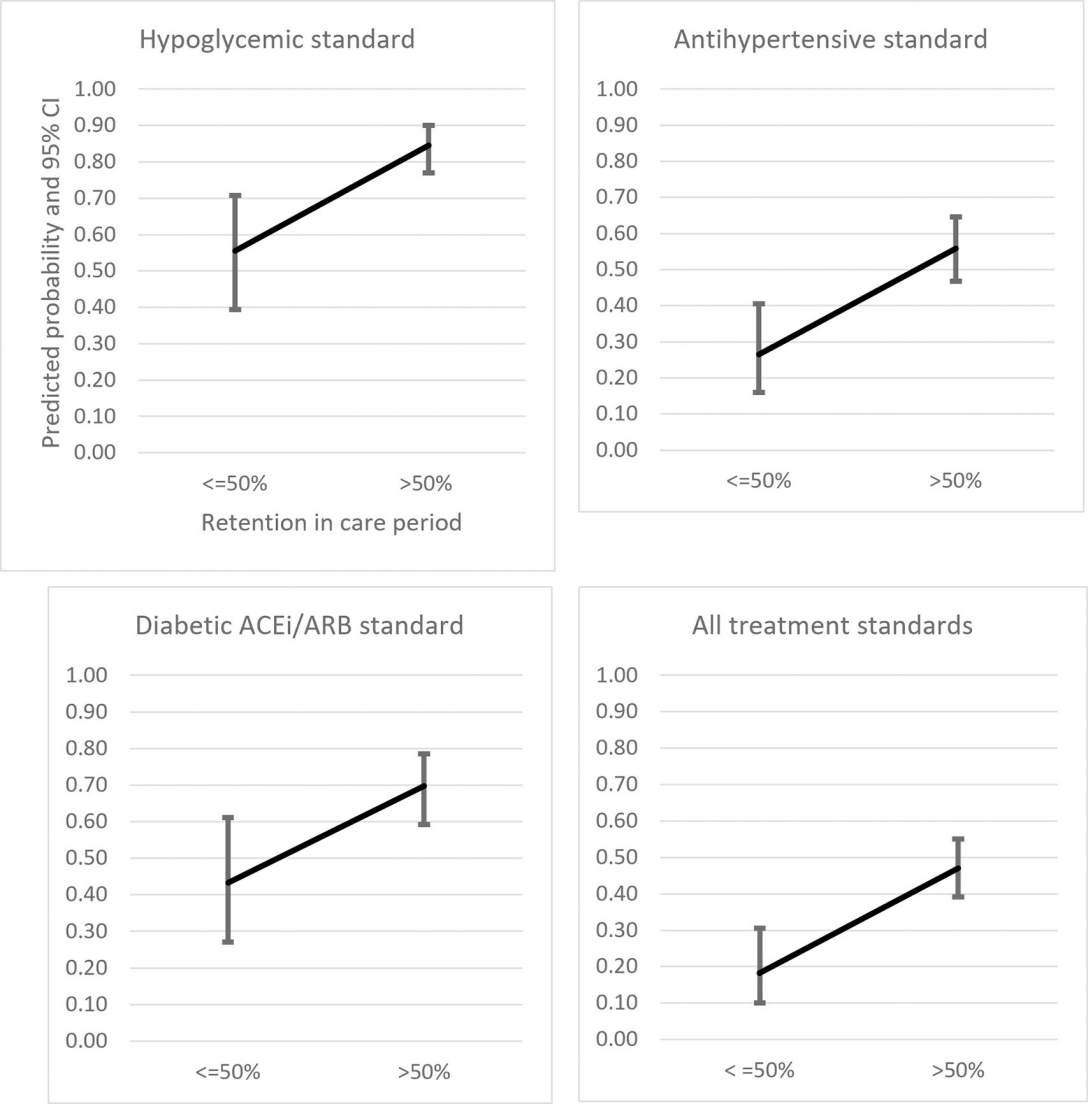
Predicted probabilities of guidelines‐based treatment standards achievement, by retention in care period (>50% versus < = 50% of allowable time for care period).

Table 3, Part A, shows the unadjusted odds ratios for ‘retention in care period’ (odds >50% relative to < = 50%, i.e., “longer” relative to “shorter”). The predicted probabilities of treatment standards achievement by retention in care (obtained from the regression coefficients) are shown in Table 3, Part B. Fig 3 is a graphical representation of this dose-response relationship. In multiple logistic regression, none of the candidate covariates for confounding or independent effects met criteria for model inclusion.

**Table 3:** Unadjusted effects of retention in care period on treatment standards achievement, exposed (>50% of allowable time), unexposed (< = 50%), N = 211.

Part A. Adjusted odds ratios (OR)			
Treatment standard met	OR	95% CI	P-value ‡
Hypoglycemic, N = 153 *	4.40	1.92–10.1	<0.001
Antihypertensive, N = 167	3.51	1.69–7.30	0.001
Diabetic ACEi/ARB, N = 116	3.02	1.28–7.11	0.011
All treatment standards, N = 204 †	3.99	1.87–8.50	0.000
Part B. Predicted probabilities (Prob) from logit coefficients			
Treatment standard met	Retention in care	Prob	95% CI
Hypoglycemic	>50%	0.85	0.77–0.90
	< = 50%	0.56	0.39–0.71
Antihypertensive	>50%	0.56	0.47–0.65
	< = 50%	0.27	0.16–0.41
Diabetic ACEi/ARB	>50%	0.70	0.59–0.79
	< = 50%	0.43	0.27–0.61
All treatment standards	>50%	0.47	0.39–0.55
	< = 50%	0.18	0.10–0.31

OR = odds ratio; 95% CI = 95% confidence interval

* 160 diabetes, 7 missing fasting glucose

† 211 (all patients) eligible, 7 missing

‡ Wald test p-value

### Retention in care (descriptive)

In the 131 post-program observations, median retention in home care was 8 months (interquartile range (IQR) 5 to 9) of an allowable 10 months. Four monthly home visits per patient were planned; the median of average monthly home visits realized was 3.2 (IQR 2.2 to 3.5). Median retention in clinic care was 13 months (IQR 9 to 14) of an allowable 17 months. One monthly clinic visit per patient was planned; the median of monthly clinic visits realized was 1.0 (IQR 0.71 to 1.33).

## Discussion

Our community-based primary care program, which incorporated four CCM elements, CHWs, and free access to care and medications (the latter from a limited formulary of seven inexpensive oral agents), was associated with greater achievement of treatment standards compared to the pre-exposure period. Further, we found no difference in treatment standards achievement between clinic and home periods. We observed a dose-response relation between retention in care and standards achievement. Longer retention, irrespective of care period, resulted in greater standards achievement.

Retention in care, measured as shorter visit intervals [54–56], continuity of care [55, 57–59], and greater visit quantity, [60] has been shown to have a positive relationship with clinical outcomes of type 2 diabetes [60] and hypertension [54, 56–58] and with treatment intensification [54, 55, 59]. Our study found a similar relationship between retention in care and an outcome measure that combined intermediate clinical outcomes and maximal treatment (the endpoint of repeated intensification).

Multi-component intervention effects on pharmacotherapy of type 2 diabetes and hypertension in LMICs have been reported in 12 studies, published between 2015 and 2023, including ten cluster-randomized trials [61–70] and two pre-post studies [23, 71]. One of these enrolled type 2 diabetics, including a subgroup with hypertension, and is the only report of effects on treatment with hypoglycemic agents [23]. Unlike the present study, effects on treatment were not primary outcomes in any (and were often reported in supplemental materials). None evaluated pharmacotherapy quality, time-bound treatment intensification, or guidelines-based protocol completion.

The single study of hypoglycemic agents found greater proportions of post-exposure diabetic patients treated with metformin and dipeptidyl peptidase 4 (DPP-4) inhibitors, when medications from three drug classes were available and cost-free to patients [23].

Of the twelve studies of multicomponent intervention effects on hypertension treatment, two found no significant between-group differences in proportions receiving “any antihypertensive treatment” [64, 65] and effects were marginal in two others [62, 70]. Even though monotherapy is ineffective in 70% to 90% of hypertensive patients [72], two studies reported a greater proportion of intervention-group patients treated with the only drug available [63, 67]. Six studies of statistical and clinical significance had follow-up of 10–36 months [23, 61, 66, 68, 69, 71]. All six reported between-group [61, 66, 68, 69] or pre-post [23, 71] differences favoring intervention or post-exposure groups, including greater mean number intensified [61], and greater proportions receiving medications (any [71], two [68], two or more [66, 69], or three or more [68]), and receiving thiazide diuretics and ACE inhibitors (or angiotensin receptor blockers (ARBs)) [23]. A greater proportion receiving two or more drug classes implies greater intensification in intervention group patients. The number of protocol-specified antihypertensive drug classes were two [69], three [61, 66], and four or more [23, 68, 71].

These studies reflect an apparent lack of capacity in LMICs, because of poor medication access and availability, to implement, from beginning to end, guidelines-based protocols for type 2 diabetes and hypertension management. Few treatment protocols had an adequate variety of medications. Among the 12 studies of multicomponent intervention effects on hypertension treatment, only seven made available at least three of the four guidelines-recommended antihypertensive drug classes [23, 61, 62, 66, 68, 70, 71]; our study made available four. The single study that enrolled patients with diabetes made available three classes of guidelines-recommended hypoglycemic drug classes [23]; our study made available only two. Access to treatment may also be a limiting factor in the studies reviewed. Payment for medications was insurance-subsidized in one study [71], out-of-pocket in five (in at least one arm or group) [63, 65–67, 69], and cost-free to all participants in two studies [23, 61].

Seven studies of clinical action measures, or the equivalent, evaluated lipid-lowering [24, 25, 32, 73, 74], antihypertensive [26, 27, 73, 74], and/or hypoglycemic [27, 73, 74] treatment in patients with diabetes and/or hypertension. One, like the present study, evaluated a clinical action measure as an outcome (the exposure was hypertension guidelines implementation strategies) [26]. One evaluated the measure as an exposure and its effects on intermediate clinical outcomes of poorly controlled diabetes, hypertension, and hyperlipidemia [73]. Five studies compared clinical action measures to traditional quality metrics [24, 25, 27, 32, 74]. In the studies reviewed, treatment intensification was evaluated within time windows, most commonly six months [25–27]. In our study, the clinical action measures were time-bound by the care period windows. Our measure captured the endpoints of glycemic or blood pressure control or, alternatively, maximal treatment. We expected these endpoints to be achieved during the care periods and, therefore, we did not account for lesser intensification.

Our patients, like others in LMICs, may be hindered in their ability to achieve glycemic targets because of the effect of late diagnosis or prolonged lack of effective therapy (“glycemic burden”) on progressive pancreatic beta-cell dysfunction. Clinical action measures would be especially useful in these settings because they account for quality of care in patients with uncontrolled glycemia and blood pressure where the greatest absolute risk reduction per increment of decline of risk factors occurs. In a retrospective cohort study of over 100,000 patients with new-onset type 2 diabetes followed for a median of 5.3 years, Paul et al [34] found that early compared to delayed treatment intensification was associated with fewer cardiovascular events even when long-term glycemic control was above the recommended threshold.

### Study strengths

Efficacy studies of multi-component interventions in LMICs have demonstrated improved clinical outcomes of type 2 diabetes and hypertension [17, 18] but they don’t evaluate how, or the mechanism by which, those outcomes were produced. When fidelity, acceptance, and feasibility and relative contributions of program components are unknown, then replication potentially wastes already-limited resources. The program in the present study was also associated with improved glycemic and blood pressure control (Table 2A). However, this was an implementation study of a process outcome (treatment standards based on repeated intensification) which has a known positive relationship to clinical outcomes [34, 36]. In turn, another process, retention in care, was associated with greater treatment standards achievement. The program had high fidelity given the proportions achieving treatment standards and the high medians of retention in care and monthly average numbers of visits.

The study program’s logic model (details in S1 Appendix) is based on the medical literature which demonstrates that care factors (retention in care, treatment intensification, out-of-pocket costs, and medication adherence) and interventions (CCM and CHWs) are inter-related and affect clinical outcomes of diabetes and hypertension. For example, in studies of diabetes and hypertension, CHWs have positive effects on retention in care [75] and retention in care improves treatment intensification [54]. CHWs [22], retention in care [54], and treatment intensification [76] are associated with improved clinical outcomes. Our study, when combined with the logic model, supports the following hypothetical pathway: CHW’s have a positive effect on retention in care, which facilitates iterative treatment intensification, which results in improved clinical outcomes. Data collection for our study ended in 2014, but the program’s methods and treatment protocols, uniqueness of an implementation (process) study, and the relative novelty of the outcome measure are as informative and useful for diabetes and hypertension care in LMICs today as then.

The study has several implications relevant to current policymaking in LMICs. The process (time-bound treatment intensification) can be measured, potentially proactively and continuously as a quality improvement tool, requiring only a limited set of widely available data, i.e., results (BP and glycemic measures), prescriptions and prescribers, and dates of results and prescriptions. A process measure, acted upon prior to poor outcomes, improves upon ad hoc quality improvement projects implemented reactively. The study program’s home care model, with its remote treatment decisions, simultaneously addresses limitations of money, manpower, and medications. A recent observational study of entirely remote protocol-driven care of hypertension in the USA had multiple components, among them computerized algorithmic decision-support, pharmacist-supervised prescriptions by CHWs, and automated remote blood pressure monitoring [77]. In a comparison of pre- and post-COVID pandemic effects, benefits significantly favored the latter, including a greater proportion with controlled blood pressure, treatment intensification, and quantities of medications, and shorter time to blood pressure control [77]. That study describes information technology (software, hardware, and telecommunications) [77, 78] which our study’s home care model lacked. Our program, however, might be easier to afford and replicate in low-resource settings. Two other studies describe apparently remote multicomponent interventions for hypertension in LMICs, one in China where CHWs prescribed [67], and another conducted in Colombia and Malaysia where CHWs recommended, and physicians prescribed, antihypertensive medication [66].

This study’s treatment protocols were intended for a low-resource setting and intentionally did not use modern pharmacotherapeutics. Thiazolidinediones, dipeptidyl peptidase-4 (DPP-4) inhibitors, glucagon-like peptide-1 (GLP-1) agonists, sodium-glucose cotransporter-2 (SGLT-2) inhibitors, and long-acting glucose analogues had been approved before 2007 (in the USA) but the program’s formulary included (and would still include) only older off-patent generic and inexpensive oral agents. The World Health Organization (WHO) added long-acting insulin analogues and SGLT-2 inhibitors to the List of Essential Medications in 2021 [79]. However, SGLT-2 inhibitors and GLP-1 agonists [80] and insulins in any form [81, 82], even older human insulins, are unavailable or inaccessible in many LMICs

The treatment protocols in the current study are comparable to the most recently published studies of multicomponent intervention effects on pharmacotherapy of type 2 diabetes and hypertension in LMICs. The only other study of multicomponent interventions effects on hypoglycemic treatment of type 2 diabetes in an LMIC, discussed above and published in 2023, also provided access only to inexpensive oral agents but, unlike the present study, included a DIPP-4 inhibitor in addition to metformin and a sulfonylurea [23]. Among the 12 studies of multicomponent interventions including effects on hypertension pharmacotherapy in LMICs (discussed above), eight were published recently (2019 to 2023) [23, 62, 65, 66, 68–71, 79] and provided access to one or more of the same four classes of antihypertensive agents used in the present study.

With minor revisions, the treatment protocols in the current study would be consistent with the most current guidelines for pharmacotherapy of type 2 diabetes and hypertension. Metformin remains the first-line hypoglycemic agent in patients with type 2 diabetes in both the American Diabetes Association (ADA) [83] and WHO guidelines [84]. However, the ADA now recommends, as first-line therapy, SGLT-2 inhibitors and/or GLP-1 agonists in patients with CVD (or high risk of CVD) or chronic kidney disease, and GLP-1 agonists to achieve weight management goals [83]. The WHO currently recommends against routine use of DPP-4 inhibitors, thiazolidinediones, GLP-1 agonists, and SGLT-2 inhibitors because of their high cost and still recommends metformin, sulfonylureas, and insulin as first-, second-, and third-line hypoglycemic agents respectively [84]. Hypertension is now defined by the American Heart Association and American College of Cardiology (AHA/ACC) [85] as SBP > = 130 or DBP > = 80 mm Hg. The WHO definition is unchanged (SBP > = 140 or DBP > = 90) [86]. Regardless of definition, the AHA/ACC [85], ADA [83], and WHO [86] all recommend pharmacotherapy for SBP > = 130 or DBP > = 80 mm Hg in patients with diabetes, or at high risk of CVD [85, 86], consistent with older guidelines (and the current study’s protocols). New recommendations include initial therapy with a two-drug combination therapy for SBP >20 or DBP >10 mm Hg above target [83, 85]; and a mineralocorticoid receptor antagonist (MRA) when BP is resistant to control with three other classes of antihypertensive agents [83, 85]. Whereas our study’s treatment protocols, based on older guidelines, used first-line ACEi or ARB for hypertension treatment in all patients with diabetes, the ADA now recommends such treatment only in patients with established coronary artery disease or albuminuria [83]. Unlike older guidelines, ADA [83] and WHO [86] now recommend low-dose aspirin only for secondary (not primary) prevention of CVD. Our study relied on home BP measurements for diagnosis and monitoring and as the basis for treatment titration. The most recent AHA/ACC [85] and European Society of Hypertension [87] guidelines now recommend out-of-office BP measurements (ambulatory or home) for diagnosis, monitoring, and medication titration.

Therefore, the present study’s treatment protocols would be made current, in the context of LMICs, by the following revisions: two-drug combinations as initial hypertension treatment in many patients, addition of an MRA for resistant hypertension, and cessation of low-dose aspirin for primary prevention of CVD in patients with diabetes. When urine albumin-to-creatinine testing is unavailable (as is still true of our setting), we recommend an ACEi or ARB for first-line treatment of elevated blood pressure in all diabetic patients.

What distinguishes the program in our study, and makes it relevant in the current context, is its intention to make best use of the few medications available. “Best use” meant “adequate titration” which requires retention in regular and frequent care. Mohan, et al, in 2020, also advocated for greater use of older inexpensive hypoglycemic agents in low-resource settings [88]. They conclude that metformin and sulfonylureas provide robust A1c reductions in diabetes but are underutilized in LMICs. A policy implication of our study is that a few low-cost medications made available to all (in ample supply to support adequate titration) may be preferable to a large variety of medications made available to fewer.

The problems this study addresses are currently relevant. The most recent data on noncommunicable disease (NCD) prevalence, financing, effective treatment coverage, and manpower in LMICs show worrisome trends, underscoring the need for primary care implemented using few resources while improving the quality of type 2 diabetes and hypertension management. Type 2 diabetes prevalence has increased and will continue to increase globally, mostly in in LMICs [8]. Only 1% of global health spending is for noncommunicable diseases (NCDs) in LMICs (where 80% of diabetics now live), even though NCDs account for 60% of global mortality [89]. Myocardial infarction and stroke account for the most disability-adjusted life years (DALYs) lost, largely attributable to type 2 diabetes and hypertension [1]. Progress in universal health coverage (UHC) has not led to more effective treatment coverage of NCDs which is outpaced by increasing NCD prevalence [90]. As a reflection of weak primary care systems, the prevalence of complications of diabetes and hypertension is increasing. In Peru during a recent 7-year period, there was a three-fold increase in chronic renal disease prevalence and a similar increase in dialysis service contracts, while the number of nephrologists per 1000 patients decreased by half [91]. By 2030, the professional healthcare workforce shortage is expected to improve only modestly (from 2013 levels) in countries that were already below the United Nation’s Sustainable Development Goal (SDG) of 4.5 professionals per 1000 population [92].

Our study demonstrates that quality evaluation should not rely solely on thresholds of intermediate clinical outcomes. 48% of study patients with diabetes would have been classified as ‘suboptimal quality’ based on glycemic control whereas clinical inertia (failure to achieve the study’s hypoglycemic treatment standard) occurred in only 20%.

### Study limitations

While the program’s methods should be reproducible in other similar settings, our results cannot be considered generalizable because they are a non-profit organization’s experience in a single community.

The program pre- versus post-exposure comparison had no control for the effect of time. A parallel comparison was also lacking for the home and clinic care periods. During the six-month hiatus between the home and clinic periods, most patients stopped filling their prescriptions, but, in the large majority that participated in both care periods, knowledge and behavioral change were presumably transferable from home to clinic and retention in care during the clinic period may have been a habit established during the earlier home care period. The absence of a difference in outcomes between clinic and home care is intriguing but the sample size had sufficient power to detect only relatively large differences. A larger sample is required to detect smaller differences which might be clinically important. Small sample size also may have prevented detection of confounding and interactions during logistic regression. However, self-matching provided confounding control in the study of the program exposure. There was partial self-matching in study of the care period exposure. 73% and 78% of home and clinic observations, respectively, were self-matched because of the large proportion of the cohort that participated in both care periods.

Longer retention in care may be a proxy for greater motivation and greater adherence to medications and other self-care behaviors. These factors may be unmeasured confounders of the association between longer retention in care and treatment standards.

HbA1c testing was unavailable to us. We used fasting glucose as a measure of glycemia, as have similar investigations [18, 93]. We minimized the effect of day-to-day variation in fasting glucose, which can be large [94], by using an aggregate value, the median of monthly average fasting glucose values. Moreover, there is a moderate [95] to strong [96–98] correlation between fasting glucose and hemoglobin A1c in patients with diabetes. Our cut-off value for defining glycemic control, 8.7 mmol/L, correlates with a hemoglobin A1c of approximately 58 mmol/mol [99]. The American Diabetes Association establishes therapeutic targets for both hemoglobin A1c and fasting glucose [43]. Fasting glucose was a therapeutic target in the seminal studies demonstrating the effect of intensive glycemic control on risk reduction in diabetes [100–102]. Hemoglobin A1c is a measure of average glucose during a 2–3-month period and, for that reason, is measured quarterly. Weekly fasting glucose values were essential for monthly treatment intensification.

## Conclusion

There are few studies of the effects of CHWs and multiple components of CCM on guideline-based pharmacotherapy of type 2 diabetes and hypertension, especially in resource-poor settings. Studies of CCM and CHW effects on longitudinal linked outcome-treatment measures, i.e., clinical action measures, and the treatment of poorly controlled patients with diabetes and hypertension are lacking. Our study helps fill that gap. The clinical action measures in this study distinguished between clinical inertia and timely treatment intensification in poorly controlled patients, a high-risk group that benefits most from treatment and constitutes a large proportion of diabetic and hypertensive patients. This implementation study also uniquely identifies relationships between multiple processes and clinical outcomes which suggest a roadmap for improvement of pharmacotherapy quality defined as guidelines-based titration of medications that are likely to be available in most LMICs. The study demonstrated the importance of retention in care, which had a dose-response relationship with achievement of treatment standards. Although our results suggest that clinic care did not improve upon home care with remote treatment decisions, a rigorous study having a parallel control group is needed to establish that with confidence. Recently reported beneficial effects of entirely remote care of hypertension in a high-income country further underscores the importance of replicating the home care model in low-resource settings.

## Supporting information

S1 Appendix Logic model for Siempre Salud primary care program for type 2 diabetes and hypertension. (PDF)

S1 File Description of Siempre Salud program of community-based primary care of type 2 diabetes and hypertension. (PDF)

S2 File STROBE statement checklist. (PDF)

S1 Table Siempre Salud standards of care for type 2 diabetes and hypertension. (PDF)

S2 Table Siempre Salud medication protocols for type 2 diabetes and hypertension. (PDF)

## References

[1] Roth GA, Mensah GA, Johnson CO, Addolorato G, Ammirati E, Baddour LM, et al. Global Burden of Cardiovascular Diseases and Risk Factors, 1990–2019: Update From the GBD 2019 Study. J Am Coll Cardiol. 2020;76(25):2982–3021. doi: 10.1016/j.jacc.2020.11.010 ; PubMed Central PMCID: PMC7755038. 33309175 PMC7755038

[2] Ettehad D, Emdin CA, Kiran A, Anderson SG, Callender T, Emberson J, et al. Blood pressure lowering for prevention of cardiovascular disease and death: a systematic review and meta-analysis. Lancet. 2016;387(10022):957–67. Epub 20151224. doi: 10.1016/S0140-6736(15)01225-8 . 26724178

[3] Adler AI, Stratton IM, Neil HA, Yudkin JA, Matthews DR, Cull CA, et al. Association of systolic blood pressure with macrovascular and microvascular complications of type 2 diabetes (UKPDS 36): prospective observational study. BMJ (Clinical research ed). 2000;321(7258):412–9. doi: 10.1136/bmj.321.7258.412 10938049 PMC27455

[4] Stratton IM, Adler A, Neil HA, Matthews DR, Manley SE, Cull CA, et al. Association of glycaemia with macrovascular and microvascular complications of type 2 diabetes (UKPDS 35): prospective observational study. BMJ (Clinical research ed). 2000;321(7258):405–12. doi: 10.1136/bmj.321.7258.405 10938048 PMC27454

[5] International Diabetes Federation. IDF Diabetes Atlas, 10th Edition, 2021: International Diabetes Foundation; 2021 [cited 2023 05/10/2023]. Available from: https://diabetesatlas.org/data/en/world/.

[6] Schutte AE, Venkateshmurthy NS, Mohan S, Prabhakaran D. Hypertension in Low- and Middle-Income Countries. Circ Res. 2021;128(7):808–26. Epub 20210401. doi: 10.1161/CIRCRESAHA.120.318729 ; PubMed Central PMCID: PMC8091106. 33793340 PMC8091106

[7] Mills KT, Stefanescu A, He J. The global epidemiology of hypertension. Nat Rev Nephrol. 2020;16(4):223–37. Epub 20200205. doi: 10.1038/s41581-019-0244-2 ; PubMed Central PMCID: PMC7998524. 32024986 PMC7998524

[8] Liu J, Bai R, Chai Z, Cooper ME, Zimmet PZ, Zhang L. Low- and middle-income countries demonstrate rapid growth of type 2 diabetes: an analysis based on Global Burden of Disease 1990–2019 data. Diabetologia. 2022;65(8):1339–52. doi: 10.1007/s00125-022-05713-6 35587275 PMC9118183

[9] Kabir A, Karim MN, Islam RM, Romero L, Billah B. Health system readiness for non-communicable diseases at the primary care level: a systematic review. BMJ Open. 2022;12(2):e060387. Epub 20220209. doi: 10.1136/bmjopen-2021-060387 ; PubMed Central PMCID: PMC8830230. 35140165 PMC8830230

[10] Bodenheimer T, Wagner EH, Grumbach K. Improving primary care for patients with chronic illness. JAMA. 2002;288(14):1775–9. doi: 10.1001/jama.288.14.1775 12365965

[11] Epping-Jordan JE, Pruitt SD, Bengoa R, Wagner EH. Improving the quality of health care for chronic conditions. Qual Saf Health Care. 2004;13(4):299–305. doi: 10.1136/qhc.13.4.299 ; PubMed Central PMCID: PMC1743863. 15289634 PMC1743863

[12] El Sayed NA, Aleppo G, Aroda VR, Bannuru RR, Brown FM, Bruemmer D, et al. 1. Improving Care and Promoting Health in Populations: Standards of Care in Diabetes—2023. Diabetes Care. 2022;46(Supplement_1):S10–S8. doi: 10.2337/dc23-S001 36507639 PMC9810463

[13] Kazemian P, Shebl FM, McCann N, Walensky RP, Wexler DJ. Evaluation of the Cascade of Diabetes Care in the United States, 2005–2016. JAMA Intern Med. 2019;179(10):1376–85. doi: 10.1001/jamainternmed.2019.2396 ; PubMed Central PMCID: PMC6692836. 31403657 PMC6692836

[14] Muntner P, Miles MA, Jaeger BC, Hannon Iii L, Hardy ST, Ostchega Y, et al. Blood Pressure Control Among US Adults, 2009 to 2012 Through 2017 to 2020. Hypertension. 2022;79(9):1971–80. Epub 20220526. doi: 10.1161/HYPERTENSIONAHA.122.19222 ; PubMed Central PMCID: PMC9370255. 35616029 PMC9370255

[15] Manne-Goehler J, Geldsetzer P, Agoudavi K, Andall-Brereton G, Aryal KK, Bicaba BW, et al. Health system performance for people with diabetes in 28 low- and middle-income countries: A cross-sectional study of nationally representative surveys. PLoS Med. 2019;16(3):e1002751. Epub 20190301. doi: 10.1371/journal.pmed.1002751 ; PubMed Central PMCID: PMC6396901. 30822339 PMC6396901

[16] Geldsetzer P, Manne-Goehler J, Marcus ME, Ebert C, Zhumadilov Z, Wesseh CS, et al. The state of hypertension care in 44 low-income and middle-income countries: a cross-sectional study of nationally representative individual-level data from 1·1 million adults. Lancet. 2019;394(10199):652–62. Epub 20190718. doi: 10.1016/s0140-6736(19)30955-9 . 31327566

[17] Goh LH, Siah CJR, Tam WWS, Tai ES, Young DYL. Effectiveness of the chronic care model for adults with type 2 diabetes in primary care: a systematic review and meta-analysis. Syst Rev. 2022;11(1):273. Epub 20221215. doi: 10.1186/s13643-022-02117-w ; PubMed Central PMCID: PMC9753411. 36522687 PMC9753411

[18] Flood D, Hane J, Dunn M, Brown SJ, Wagenaar BH, Rogers EA, et al. Health system interventions for adults with type 2 diabetes in low- and middle-income countries: A systematic review and meta-analysis. PLoS Med. 2020;17(11):e1003434. Epub 20201112. doi: 10.1371/journal.pmed.1003434 ; PubMed Central PMCID: PMC7660583. 33180775 PMC7660583

[19] Kim K, Choi JS, Choi E, Nieman CL, Joo JH, Lin FR, et al. Effects of Community-Based Health Worker Interventions to Improve Chronic Disease Management and Care Among Vulnerable Populations: A Systematic Review. Am J Public Health. 2016;106(4):e3–e28. Epub 20160218. doi: 10.2105/AJPH.2015.302987 ; PubMed Central PMCID: PMC4785041. 26890177 PMC4785041

[20] Brownstein JN, Chowdhury FM, Norris SL, Horsley T, Jack L, Jr., Zhang X, et al. Effectiveness of community health workers in the care of people with hypertension. Am J Prev Med. 2007;32(5):435–47. doi: 10.1016/j.amepre.2007.01.011 . 17478270

[21] Anand TN, Joseph LM, Geetha AV, Prabhakaran D, Jeemon P. Task sharing with non-physician health-care workers for management of blood pressure in low-income and middle-income countries: a systematic review and meta-analysis. The Lancet Global Health. 2019;7(6):e761–e71. doi: 10.1016/S2214-109X(19)30077-4 31097278 PMC6527522

[22] Palmas W, March D, Darakjy S, Findley SE, Teresi J, Carrasquillo O, et al. Community Health Worker Interventions to Improve Glycemic Control in People with Diabetes: A Systematic Review and Meta-Analysis. J Gen Intern Med. 2015;30(7):1004–12. doi: 10.1007/s11606-015-3247-0 25735938 PMC4471021

[23] Silva-Tinoco R, Cuatecontzi-Xochitiotzi T, Castillo-Martínez L, De La Torre-Saldaña V, Guzman-Olvera E, Bernal-Ceballos F. Impact of a multicomponent integrated care delivery program on diabetes care goals achievement: a primary care quality improvement initiative. Prim Care Diabetes. 2023;17(6):568–74. doi: 10.1016/j.pcd.2023.07.004 37640623

[24] Beard AJ, Hofer TP, Downs JR, Lucatorto M, Klamerus ML, Holleman R, et al. Assessing Appropriateness of Lipid Management Among Patients With Diabetes Mellitus. Circ Cardiovasc Qual Outcomes. 2013;6(1):66–74. doi: 10.1161/circoutcomes.112.966697 23233749 PMC3699178

[25] Kerr EA, Smith DM, Hogan MM, Hofer TP, Krein SL, Bermann M, et al. Building a Better Quality Measure: Are Some Patients With ‘Poor Quality’ Actually Getting Good Care? Med Care. 2003;41(10). doi: 10.1097/01.MLR.0000088453.57269.29 14515113

[26] Green LA, Wyszewianski L, Lowery JC, Kowalski CP, Krein SL. An observational study of the effectiveness of practice guideline implementation strategies examined according to physicians’ cognitive styles. Implementation Science. 2007;2(1):41. doi: 10.1186/1748-5908-2-41 18053156 PMC2219964

[27] Sidorenkov G, Haaijer-Ruskamp FM, de Zeeuw D, Denig P. A longitudinal study examining adherence to guidelines in diabetes care according to different definitions of adequacy and timeliness. PLoS One. 2011;6(9):e24278. Epub 20110908. doi: 10.1371/journal.pone.0024278 ; PubMed Central PMCID: PMC3169586. 21931669 PMC3169586

[28] Voorham J, Haaijer-Ruskamp FM, Van Der Meer K, De Zeeuw D, Wolffenbuttel BHR, Hoogenberg K, et al. Identifying targets to improve treatment in type 2 diabetes; the Groningen Initiative to aNalyse Type 2 diabetes Treatment (GIANTT) observational study. Pharmacoepidemiol Drug Saf. 2010;19(10):1078–86. doi: 10.1002/pds.2023 20687048

[29] Chiu N, Chiu L, Aggarwal R, Raber I, Bhatt DL, Mukamal KJ. Trends in Blood Pressure Treatment Intensification in Older Adults With Hypertension in the United States, 2008 to 2018. Hypertension. 2023;80(3):553–62. doi: 10.1161/HYPERTENSIONAHA.122.19882 36111537

[30] Khunti K, Seidu S. Therapeutic Inertia and the Legacy of Dysglycemia on the Microvascular and Macrovascular Complications of Diabetes. Diabetes Care. 2019;42(3):349–51. doi: 10.2337/dci18-0030 30787057

[31] Jiang DH, O’Connor PJ, Huguet N, Golden SH, McCoy RG. Modernizing Diabetes Care Quality Measures. Health Aff (Millwood). 2022;41(7):955–62. Epub 20220627. doi: 10.1377/hlthaff.2022.00233 ; PubMed Central PMCID: PMC9288231. 35759700 PMC9288231

[32] Lavens A, Doggen K, Mathieu C, Nobels F, Vandemeulebroucke E, Vandenbroucke M, et al. Clinical action measures improve the reliability of feedback on quality of care in diabetes centres: a retrospective cohort study. BMC Health Serv Res. 2016;16(1). doi: 10.1186/s12913-016-1670-5 27553193 PMC4995611

[33] Kaewbut P, Kosachunhanun N, Phrommintikul A, Chinwong D, Hall JJ, Chinwong S. Time to Treatment Intensification to Reduce Diabetes-Related Complications: A Post Hoc Study. Healthcare. 2022;10(9):1673. doi: 10.3390/healthcare10091673 36141285 PMC9498838

[34] Paul SK, Klein K, Thorsted BL, Wolden ML, Khunti K. Delay in treatment intensification increases the risks of cardiovascular events in patients with type 2 diabetes. Cardiovasc Diabetol. 2015;14:100. Epub 20150807. doi: 10.1186/s12933-015-0260-x ; PubMed Central PMCID: PMC4528846. 26249018 PMC4528846

[35] Paldánius PM. Evaluating the Evidence behind the Novel Strategy of Early Combination from Vision to Implementation. Diabetes Metab J. 2020;44(6):785–801. doi: 10.4093/dmj.2020.0179 33081426 PMC7801764

[36] Sidorenkov G, Haaijer-Ruskamp FM, de Zeeuw D, Bilo H, Denig P. Review: Relation Between Quality-of-Care Indicators for Diabetes and Patient Outcomes: A Systematic Literature Review. Med Care Res Rev. 2011;68(3):263–89. doi: 10.1177/1077558710394200 . 21536606

[37] von Elm E, Altman DG, Egger M, Pocock SJ, Gøtzsche PC, Vandenbroucke JP. The Strengthening the Reporting of Observational Studies in Epidemiology (STROBE) statement: guidelines for reporting observational studies. The Lancet. 2007;370(9596):1453–7. doi: 10.1016/S0140-6736(07)61602-X 18064739

[38] Deaver JE. Community-based Primary Care Program Effects on Pharmacotherapy of Type 2 Diabetes and Hypertension in Peru. ClinicalTrials.gov identifier: NCT05979142. 2023 [updated 08/27/2023; cited 2023 08/27/2023]. Available from: https://www.clinicaltrials.gov/study/NCT05979142.

[39] World Bank Poverty and Equity Brief, Latin America and the Caribbean, Peru, April 2021. Available from: https://databank.worldbank.org/data/download/poverty/987B9C90-CB9F-4D93-AE8C-750588BF00QA/AM2020/Global_POVEQ_PER.pdf.

[40] Deaver JE. Prospective cohort study of a community-based primary care program’s effects on pharmacotherapy quality in low-income Peruvians with type 2 diabetes and hypertension [Dataset]. Dryad. 2023. doi: 10.5061/dryad.76hdr7t1n PMC11341050 39173046

[41] Naurez J, Torres J. IMF Working Paper. Unintended Effects From the Expansion of the Non-Contributory Health System in Peru. Washington, DC, USA: International Monetary Fund, 2021.

[42] Funnell MM, Brown TL, Childs BP, Haas LB, Hosey GM, Jensen B, et al. National standards for diabetes self-management education. Diabetes Care. 2007;30(6):1630–7. doi: 10.2337/dc07-9923 17526822

[43] American Diabetes Association. Standards of Medical Care in Diabetes—2011. Diabetes Care. 2011;34(Supplement 1):S11–S61. doi: 10.2337/DC11-S011 21193625 PMC3006050

[44] American Association of Diabetes Educators. AADE 7 Self-Care Behaviors American Association of Diabetes Educators (AADE) Position Statement Introduction. 2011. [Cited on 07/18/24]. Available from: https://diabetesed.net/wp-content/uploads/2022/05/aade7-self-care-behaviors-position-statement.pdf.

[45] American Diabetes Association. Nutrition Recommendations and Interventions for Diabetes. Diabetes Care. 2008;31(Supplement 1):S61–S78. doi: 10.2337/DC08-S061 18165339

[46] Nathan DM, Buse JB, Davidson MB, Ferrannini E, Holman RR, Sherwin R, et al. Medical Management of Hyperglycemia in Type 2 Diabetes: A Consensus Algorithm for the Initiation and Adjustment of Therapy. Diabetes Care. 2009;32(1):193–203. doi: 10.2337/DC08-9025 18945920 PMC2606813

[47] World Health Orgnization. Package of Essential Noncommunicable (PEN) Disease Interventions for Primary Health Care in Low-Resource Settings. Geneva, Switzerland: World Health Organization; 2010. 1–66 p.

[48] World Health Organization. Prevention of cardiovascular disease. Guidelines for the assessment and management of total cardiovascular risk. Geneva, Switzerland: World Health Organization, 2007.

[49] Chobanian AV, Bakris GL, Black HR, Cushman WC, Green LA, Izzo JL, Jr., et al. The Seventh Report of the Joint National Committee on Prevention, Detection, Evaluation, and Treatment of High Blood Pressure: the JNC 7 report. JAMA. 2003;289(19):2560–72. Epub 20030514. doi: 10.1001/jama.289.19.2560 . 12748199

[50] Pearson TA, Blair SN, Daniels SR, Eckel RH, Fair JM, Fortmann SP, et al. AHA Guidelines for Primary Prevention of Cardiovascular Disease and Stroke: 2002 Update: Consensus Panel Guide to Comprehensive Risk Reduction for Adult Patients Without Coronary or Other Atherosclerotic Vascular Diseases. American Heart Association Science Advisory and Coordinating Committee. Circulation. 2002;106(3):388–91. doi: 10.1161/01.cir.0000020190.45892.75 . 12119259

[51] D’Agostino RB, Vasan RS, Pencina MJ, Wolf PA, Cobain M, Massaro JM, et al. General cardiovascular risk profile for use in primary care: The Framingham heart study. Circulation. 2008;117(6):743–53. doi: 10.1161/CIRCULATIONAHA.107.699579 18212285

[52] World Health Organization. The Global Health Observatory. Obesity. Geneva, Switzerland: World Health Organization; [cited 2023 08/30/2023]. Available from: https://www.who.int/data/gho/indicator-metadata-registry/imr-details/3420.

[53] Inlow M. How do I obtain confidence intervals for the predicted probabilities after logistic regression?: StataCorp; [05/12/2023]. Available from: https://www.stata.com/support/faqs/statistics/prediction-confidence-intervals/.

[54] Morrison F, Shubina M, Turchin A. Encounter frequency and serum glucose level, blood pressure, and cholesterol level control in patients with diabetes mellitus. Arch Intern Med. 2011;171(17):1542–50. doi: 10.1001/archinternmed.2011.400 ; PubMed Central PMCID: PMC3692291. 21949161 PMC3692291

[55] Turchin A, Goldberg SI, Shubina M, Einbinder JS, Conlin PR. Encounter frequency and blood pressure in hypertensive patients with diabetes mellitus. Hypertension. 2010;56(1):68–74. Epub 20100524. doi: 10.1161/HYPERTENSIONAHA.109.148791 ; PubMed Central PMCID: PMC3752696. 20497991 PMC3752696

[56] Xu W, Goldberg SI, Shubina M, Turchin A. Optimal systolic blood pressure target, time to intensification, and time to follow-up in treatment of hypertension: population based retrospective cohort study. BMJ. 2015;350:h158. Epub 20150205. doi: 10.1136/bmj.h158 ; PubMed Central PMCID: PMC4353282. 25655523 PMC4353282

[57] Barrera L, Oviedo D, Silva A, Tovar D, Méndez F. Continuity of Care and the Control of High Blood Pressure at Colombian Primary Care Services. Inquiry. 2021;58:469580211047043. doi: 10.1177/00469580211047043 ; PubMed Central PMCID: PMC8511938. 34620003 PMC8511938

[58] Chan KS, Wan EY, Chin WY, Cheng WH, Ho MK, Yu EY, et al. Effects of continuity of care on health outcomes among patients with diabetes mellitus and/or hypertension: a systematic review. BMC Fam Pract. 2021;22(1):145. Epub 20210703. doi: 10.1186/s12875-021-01493-x ; PubMed Central PMCID: PMC8254900. 34217212 PMC8254900

[59] Jackson C, Ball L. Continuity of care: Vital, but how do we measure and promote it? Aust J Gen Pract. 2018;47(10):662–4. doi: 10.31128/AJGP-05-18-4568 . 31195766

[60] Reininger BM, Lopez J, Zolezzi M, Lee M, Mitchell-Bennett LA, Xu T, et al. Participant engagement in a community health worker-delivered intervention and type 2 diabetes clinical outcomes: a quasiexperimental study in MexicanAmericans. BMJ Open. 2022;12(11):e063521. doi: 10.1136/bmjopen-2022-063521 36446462 PMC9710373

[61] He J, Irazola V, Mills KT, Poggio R, Beratarrechea A, Dolan J, et al. Effect of a Community Health Worker-Led Multicomponent Intervention on Blood Pressure Control in Low-Income Patients in Argentina: A Randomized Clinical Trial. JAMA. 2017;318(11):1016–25. doi: 10.1001/jama.2017.11358 ; PubMed Central PMCID: PMC5761321. 28975305 PMC5761321

[62] Jafar TH, Gandhi M, De Silva HA, Jehan I, Naheed A, Finkelstein EA, et al. A Community-Based Intervention for Managing Hypertension in Rural South Asia. N Engl J Med. 2020;382(8):717–26. doi: 10.1056/NEJMoa1911965 32074419

[63] Mendis S, Johnston SC, Fan W, Oladapo O, Cameron A, Faramawi MF. Cardiovascular risk management and its impact on hypertension control in primary care in low-resource settings: a cluster-randomized trial. Bull World Health Organ. 2010;88(6):412–9. doi: 10.2471/BLT.08.062364 20539854 PMC2878142

[64] Neupane D, McLachlan CS, Mishra SR, Olsen MH, Perry HB, Karki A, et al. Effectiveness of a lifestyle intervention led by female community health volunteers versus usual care in blood pressure reduction (COBIN): an open-label, cluster-randomised trial. The Lancet Global Health. 2018;6(1):e66–e73. doi: 10.1016/S2214-109X(17)30411-4 29241617

[65] Peiris D, Praveen D, Mogulluru K, Ameer MA, Raghu A, Li Q, et al. SMARThealth India: A stepped-wedge, cluster randomised controlled trial of a community health worker managed mobile health intervention for people assessed at high cardiovascular disease risk in rural India. PLoS One. 2019;14(3):e0213708. doi: 10.1371/journal.pone.0213708 30913216 PMC6435227

[66] Schwalm JD, McCready T, Lopez-Jaramillo P, Yusoff K, Attaran A, Lamelas P, et al. A community-based comprehensive intervention to reduce cardiovascular risk in hypertension (HOPE 4): a cluster-randomised controlled trial. Lancet. 2019;394(10205):1231–42. doi: 10.1016/S0140-6736(19)31949-X 31488369

[67] Tian M, Ajay VS, Dunzhu D, Hameed SS, Li X, Liu Z, et al. A Cluster-Randomized, Controlled Trial of a Simplified Multifaceted Management Program for Individuals at High Cardiovascular Risk (SimCard Trial) in Rural Tibet, China, and Haryana, India. Circulation. 2015;132(9):815–24. doi: 10.1161/CIRCULATIONAHA.115.015373 26187183 PMC4558306

[68] Wang Z, Wang X, Shen Y, Li S, Chen Z, Zheng C, et al. Effect of a Workplace-Based Multicomponent Intervention on Hypertension Control: A Randomized Clinical Trial. JAMA Cardiology. 2020;5(5):567–75. doi: 10.1001/jamacardio.2019.6161 32129791 PMC7057176

[69] Wei X, Zhang Z, Chong MKC, Hicks JP, Gong W, Zou G, et al. Evaluation of a package of risk-based pharmaceutical and lifestyle interventions in patients with hypertension and/or diabetes in rural China: A pragmatic cluster randomised controlled trial. PLoS Med. 2021;18(7):e1003694. doi: 10.1371/journal.pmed.1003694 34197452 PMC8284676

[70] Zhou H, Wang X, Yang Y, Chen Z, Zhang L, Zheng C, et al. Effect of a Multicomponent Intervention Delivered on a Web-Based Platform on Hypertension Control: A Cluster Randomized Clinical Trial. JAMA Netw Open. 2022;5(12):e2245439. Epub 20221201. doi: 10.1001/jamanetworkopen.2022.45439 ; PubMed Central PMCID: PMC9856259. 36477479 PMC9856259

[71] Valdés González Y, Campbell NRC, Pons Barrera E, Calderón Martínez M, Pérez Carrera A, Morales Rigau JM, et al. Implementation of a community‐based hypertension control program in Matanzas, Cuba. The Journal of Clinical Hypertension. 2020;22(2):142–9. doi: 10.1111/jch.13814 31967722 PMC8029874

[72] Gradman AH, Basile JN, Carter BL, Bakris GL. Combination Therapy in Hypertension. The Journal of Clinical Hypertension. 2011;13(3):146–54. doi: 10.1111/j.1751-7176.2010.00397.x 21366845 PMC8673364

[73] Selby JV, Uratsu CS, Fireman B, Schmittdiel JA, Peng T, Rodondi N, et al. Treatment Intensification and Risk Factor Control. Med Care. 2009;47(4):395–402. doi: 10.1097/mlr.0b013e31818d775c 19330888 PMC4118744

[74] Voorham J, Denig P, Wolffenbuttel BHR, Haaijer-Ruskamp FM. Cross-Sectional Versus Sequential Quality Indicators of Risk Factor Management in Patients with Type 2 Diabetes. Med Care. 2008;46(2):133–41. doi: 10.1097/MLR.0b013e31815b9da0 18219241

[75] Mbuthia GW, Magutah K, Pellowski J. Approaches and outcomes of community health worker’s interventions for hypertension management and control in low-income and middle-income countries: systematic review. BMJ Open. 2022;12(4):e053455. Epub 20220401. doi: 10.1136/bmjopen-2021-053455 ; PubMed Central PMCID: PMC8977767. 35365519 PMC8977767

[76] Desai U, Kirson NY, Kim J, Khunti K, King S, Trieschman E, et al. Time to Treatment Intensification After Monotherapy Failure and Its Association With Subsequent Glycemic Control Among 93,515 Patients With Type 2 Diabetes. Diabetes Care. 2018;41(10):2096–104. Epub 20180821. doi: 10.2337/dc17-0662 . 30131396

[77] Lee SG, Blood AJ, Cannon CP, Gordon WJ, Nichols H, Zelle D, et al. Remote Cardiovascular Hypertension Program Enhanced Blood Pressure Control During the COVID‐19 Pandemic. Journal of the American Heart Association. 2023;12(6). doi: 10.1161/JAHA.122.027296 36915035 PMC10111523

[78] Fisher NDL, Fera LE, Dunning JR, Desai S, Matta L, Liquori V, et al. Development of an entirely remote, non‐physician led hypertension management program. Clin Cardiol. 2019;42(2):285–91. doi: 10.1002/clc.23141 30582181 PMC6712321

[79] World Health Organization. WHO Model List of Essential Medicines 22nd list 2021. Geneva: World Health Organization, 2021.

[80] Barber MJ, Gotham D, Bygrave H, Cepuch C. Estimated Sustainable Cost-Based Prices for Diabetes Medicines. JAMA Network Open. 2024;7(3):e243474–e. doi: 10.1001/jamanetworkopen.2024.3474 38536176 PMC10973901

[81] Ewen M, Joosse H-J, Beran D, Laing R. Insulin prices, availability and affordability in 13 low-income and middle-income countries. BMJ Global Health. 2019;4(3):e001410. doi: 10.1136/bmjgh-2019-001410 31263585 PMC6570978

[82] Chow CK, Ramasundarahettige C, Hu W, AlHabib KF, Avezum A, Cheng X, et al. Availability and affordability of essential medicines for diabetes across high-income, middle-income, and low-income countries: a prospective epidemiological study. The Lancet Diabetes & Endocrinology. 2018;6(10):798–808. doi: 10.1016/S2213-8587(18)30233-X 30170949

[83] American Diabetes Association Professional Practice Committee. Standards of Care in Diabetes—2024. Diabetes Care. 2023;47(Supplement 1):S1–S321. doi: 10.2337/dc24-SINT

[84] World Health Organization. Diagnosis and management of type 2 diabetes (HEARTS-D). Geneva: World Health Organization, 2020.

[85] Whelton PK, Carey RM, Aronow WS, Casey DE, Collins KJ, Dennison Himmelfarb C, et al. 2017 ACC/AHA/AAPA/ABC/ACPM/AGS/APhA/ASH/ASPC/NMA/PCNA Guideline for the Prevention, Detection, Evaluation, and Management of High Blood Pressure in Adults: A Report of the American College of Cardiology/American Heart Association Task Force on Clinical Practice Guidelines. J Am Coll Cardiol. 2018;71(19):e127–e248. doi: 10.1016/j.jacc.2017.11.006 29146535

[86] World Health Organization. HEARTS technical package for cardiovascular disease management in primary health care: evidence-based treatment protocols. Geneva: World Health Organization, 2018.

[87] Stergiou GS, Palatini P, Parati G, O’Brien E, Januszewicz A, Lurbe E, et al. 2021 European Society of Hypertension practice guidelines for office and out-of-office blood pressure measurement. J Hypertens. 2021;39(7). doi: 10.1097/HJH.0000000000002843 33710173

[88] Mohan V, Khunti K, Chan SP, Filho FF, Tran NQ, Ramaiya K, et al. Management of Type 2 Diabetes in Developing Countries: Balancing Optimal Glycaemic Control and Outcomes with Affordability and Accessibility to Treatment. Diabetes Ther. 2020;11(1):15–35. doi: 10.1007/s13300-019-00733-9 31773420 PMC6965543

[89] Kodali PB. Achieving Universal Health Coverage in Low- and Middle-Income Countries: Challenges for Policy Post-Pandemic and Beyond. Risk Manag Healthc Policy. 2023;Volume 16:607–21. doi: 10.2147/RMHP.S366759 37050920 PMC10084872

[90] Lozano R, Fullman N, Mumford JE, Knight M, Barthelemy CM, Abbafati C, et al. Measuring universal health coverage based on an index of effective coverage of health services in 204 countries and territories, 1990–2019: a systematic analysis for the Global Burden of Disease Study 2019. The Lancet. 2020;396(10258):1250–84. doi: 10.1016/s0140-6736(20)30750-9 32861314 PMC7562819

[91] Herrera-Añazco P, Atamari-Anahui N, Flores-Benites V. Número de nefrólogos, servicios de hemodiálisis y tendencia de la prevalencia de enfermedad renal crónica en el Ministerio de Salud de Perú. Rev Peru Med Exp Salud Publica. 2019;36(1):62. doi: 10.17843/rpmesp.2019.361.4253 31116340

[92] World Health Organization. Global strategy on human resources for health: Workforce 2030. Geneva, Switzerland: World Health Organization; 2016.

[93] Maria JL, Anand TN, Dona B, Prinu J, Prabhakaran D, Jeemon P. Task-sharing interventions for improving control of diabetes in low-income and middle-income countries: a systematic review and meta-analysis. The Lancet Global Health. 2021;9(2):e170–e80. doi: 10.1016/S2214-109X(20)30449-6 33242455 PMC8279953

[94] Sacks DB. A1C Versus Glucose Testing: A Comparison. Diabetes Care. 2011;34(2):518–. doi: 10.2337/dc10-1546 21270207 PMC3024379

[95] Alzahrani N, Alouffi S, Almutairi K, Almutairi M, Almutairi T, Alwan IA, et al. Can fasting blood sugar be used as an indicator of long-term diabetic control instead of estimated average glucose? Clinical Laboratory. 2020;66(12):2469–74. doi: 10.7754/Clin.Lab.2020.200324 33337826

[96] Van ’t Riet E, Alssema M, Rijkelijkhuizen JM, Kostense PJ, Nijpels G, Dekker JM. Relationship between A1C and glucose levels in the general Dutch population: the new Hoorn study. Diabetes Care. 2010;33(1):61–6. doi: 10.2337/dc09-0677 19808928 PMC2797987

[97] Ramachandran A, Riddle MC, Kabali C, Gerstein HC. Relationship between A1C and fasting plasma glucose in dysglycemia or type 2 diabetes: an analysis of baseline data from the ORIGIN trial. Diabetes Care. 2012;35(4):749–53. doi: 10.2337/dc11-1918 22323416 PMC3308289

[98] Bozkaya G, Ozgu E, Karaca B. The association between estimated average glucose levels and fasting plasma glucose levels. Clinics (Sao Paulo, Brazil). 2010;65(11):1077–80. doi: 10.1590/s1807-59322010001100003 21243275 PMC2999698

[99] Wei N, Zheng H, Nathan DM. Empirically establishing blood glucose targets to achieve HbA1c goals. Diabetes Care. 2014;37(4):1048–51. doi: 10.2337/dc13-2173 24513588 PMC3964488

[100] Nathan DM, Genuth S, Lachin J, Cleary P, Crofford O, Davis M, et al. The effect of intensive treatment of diabetes on the development and progression of long-term complications in insulin-dependent diabetes mellitus. N Engl J Med. 1993;329(14):977–86. doi: 10.1056/NEJM199309303291401 . 8366922

[101] U. K. Prospective Diabetes Study Group. Intensive blood-glucose control with sulphonylureas or insulin compared with conventional treatment and risk of complications in patients with type 2 diabetes (UKPDS 33). The Lancet. 1998;352(9131):837–53. doi: 10.1016/S0140-6736(98)07019-6 9742976

[102] Diabetes Control Complications Trial/ Epidemiology of Diabetes Interventions Complications Study Research Group. Intensive diabetes treatment and cardiovascular disease in patients with type 1 diabetes. The New England journal of medicine. 2005;353(25):2643–53. doi: 10.1056/NEJMoa052187 16371630 PMC2637991

