# Supplementary material for: A retrospective cohort study of a community-based primary care program’s effects on pharmacotherapy quality in low-income Peruvians with type 2 diabetes and hypertension
Source: PLOS Glob Public Health. 2024 Aug 22;4(8):e0003512. doi: 10.1371/journal.pgph.0003512 (PMC11341050; doi:10.1371/journal.pgph.0003512)
Supplement: S1 Appendix — (PDF) [file pgph.0003512.s001.pdf]

Appendix S1. Logic Model of Siempre Salud Primary Care Program for Chronic Diseases

In patients with diabetes and hypertension, treatment inertia (failure to intensify when indicated) (1-3), medication cost burden (4-6), and poor medication adherence (7, 8) are prevalent in both high-income countries (2, 3, 7) and LMICs (1, 4-6, 8).

The primary care program is based on a logic model with inter-related processes, each associated with clinical outcomes (Figure 1 and Table 1), and the effect of interventions (the Chronic Care Model (CCM), community health workers (CHWs), and reduced out-of-pocket (OOP) costs) on these processes and outcomes (Figure 2 and Table 2). The program specifically targets the process triad of retention in care, OOP costs, and treatment intensification.

Figure 1. Inter-related care processes and clinical outcomes of type 2 diabetes and hypertension

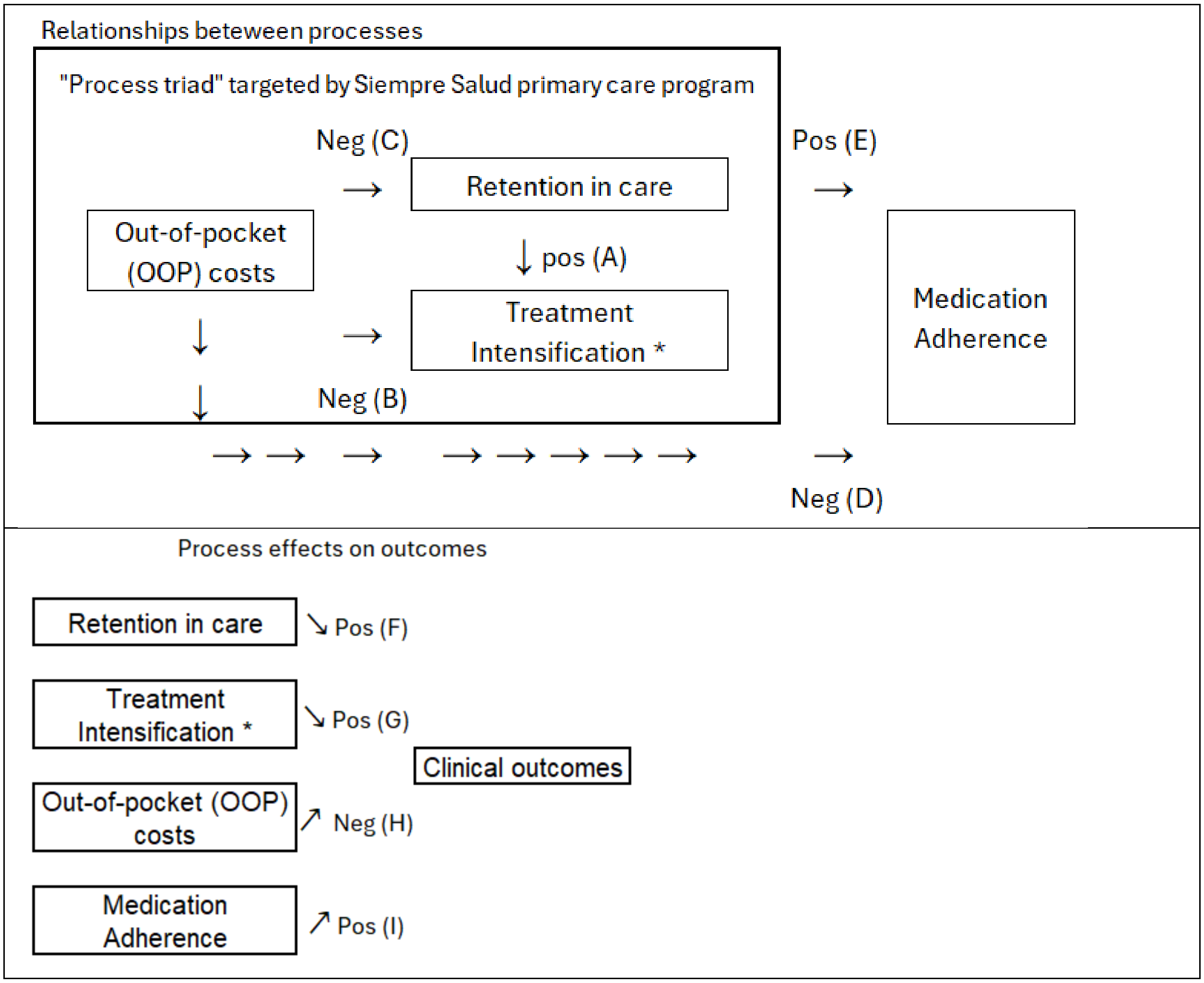

Figure 1 footnotes:
* Treatment intensification is defined as initiation or a switch, dosage increase, or addition of medication.
(A) Retention in care, e.g., shorter visit intervals (9, 10) and continuity of care (10, 11), has positive effects on treatment intensification (9, 10) and mitigates treatment inertia (12).
(B) Out-of-pocket medication costs are associated with treatment inertia (13, 14).
(C) Out-of-pocket medication costs are associated with poor retention in care (13-15),
(D) Out-of-pocket medication costs are associated with poor medication adherence (16-19).

(E) Retention in care, e.g., shorter visit intervals (9, 10) and continuity of care (10, 11) improves medication adherence (9-11).

(F) Retention in care associated with intermediate and long-term clinical outcomes of diabetes and hypertension ((9, 20-22).

(G) Treatment intensification associated with clinical outcomes (13, 23-27).

(H) OOP costs have negative association with clinical outcomes (19).

(I) Medication adherence associated with clinical outcomes of diabetes and hypertension and other illnesses (16) (Eaddy 2012).

(F) to (I). Also see “Table 2. Care factor associations with clinical outcomes”.

Table 1. Process associations with clinical outcomes of type 2 diabetes and hypertension

Author	Process	Measure	Term	DM	HTN	Oher
Morrison 2011 (9)	Retention in care	Visit interval	Intermediate	X	X	
Barrera 2021 (20)	Retention in care	COC	Intermediate		X	
Chan 2021 (21)	Retention in care	COC	Long-term	X	X	
Xu 2015 (22)	Retention in care	Visit interval	Long-term		X	
Pantalone 2016 (23)	Treatment inertia		Intermediate	X		
Desai 2018 (24)	Treatment inertia		Intermediate	X		
Arnold 2018 (25)	Treatment inertia		Intermediate	X		
Maddox 2010 (26)	Treatment inertia		Intermediate, long-term		X	
Paul 2015 (27)	Treatment inertia		Long-term	X		
Khunti 2019 (13)	Treatment inertia		Intermediate, long-term	X		
Eaddy 2012 (16)	Adherence		Intermediate	X	X	X
Hsu 2006 (19)	OOP costs		Intermediate, long-term	X	X	

DM = type 2 diabetes, HTN = hypertension

Figure 2 and Table 2 shows CCM, CHW, and reduced OOP cost interventions (the three elements of the Siempre Salud primary care program for chronic diseases) and their effects on processes and outcomes.

Figure 2. Intervention associations with improved processes and clinical outcomes

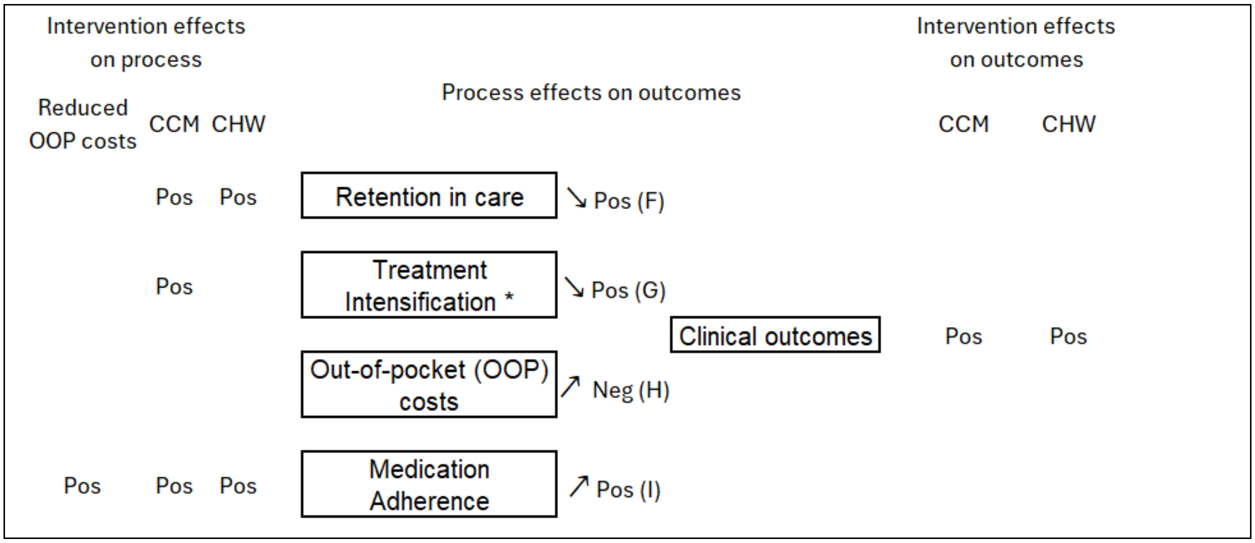

Appendix S1. Logic Model of Siempre Salud Primary Care Program for Chronic Diseases

Table 2. Interventions associated with improved processes and clinical outcomes

Process/Outcome	CCM †	CHW ‡	Reduced OOP costs §
Retention in care	X	X	
Treatment intensification	X		
Medication adherence	X	X	X
Clinical outcomes	X	X	

Table 2. Footnotes:
CCM = two or more elements of the Chronic Care Model, CHW = community health worker, OOP = out-of-pocket

† Systematic reviews have found evidence, in both HICs (28) and LMICs (29), for beneficial effects of CCM on intermediate clinical outcomes of type 2 diabetes and hypertension. Multiple components of CCM have positive effects on hemoglobin A1c (HbA1c) or fasting glucose reduction or glycemic control (28, 29), and reduced or controlled blood pressure (28), in patients with diabetes. CCM elements, or literature descriptions than can be categorized as such, have positive associations with retention in care (30-33), treatment intensification (13, 14, 34-38) and medication adherence (39, 40).

‡ Systematic reviews have also found evidence for positive effects of CHWs, especially when delivering self-management education and support, on reduced or controlled blood pressure in patients with hypertension, in both HICs (41, 42) and LMICs (43); and on HbA1c reduction or glycemic control in patients with diabetes, in HICs (44). A meta-analysis found no effect of CHWs on HbA1c or fasting glucose reduction in LMICs (45). CHWs have positive effects on retention in care (42, 46-50), and medication adherence (42, 51, 52).

§ Studies of policies that reduce out-of-pocket medication costs in high-income countries uniformly find improved medication adherence (39, 53).

References:

1. Wan KS, Moy FM, Mohd Yusof K, Mustapha FI, Mohd Ali Z, Hairi NN. Clinical inertia in type 2 diabetes management in a middle-income country: A retrospective cohort study. PLoS One. 2020;15(10):e0240531.
2. Fu AZ, Qiu Y, Davies MJ, Radican L, Engel SS. Treatment intensification in patients with type 2 diabetes who failed metformin monotherapy. Diabetes Obes Metab. 2011;13(8):765-9.
3. Khunti K, Godec TR, Medina J, Garcia-Alvarez L, Hiller J, Gomes MB, et al. Patterns of glycaemic control in patients with type 2 diabetes mellitus initiating second-line therapy after metformin monotherapy: Retrospective data for 10 256 individuals from the United Kingdom and Germany. Diabetes Obes Metab. 2018;20(2):389-99.
4. Hernández-Vásquez A, Vargas-Fernández R, Magallanes-Quevedo L, Bendezu-Quispe G. [Out-of-pocket expenditure on medicines and supplies in Peru in 2007 and 2016]. Medwave. 2020;20(2):e7833.
5. Hernández-Vásquez A, Rojas-Roque C, Vargas-Fernández R, Rosselli D. Measuring Out-of-pocket Payment, Catastrophic Health Expenditure and the Related Socioeconomic Inequality in Peru: A Comparison Between 2008 and 2017. J Prev Med Public Health. 2020;53(4):266-74.
6. Schutte AE, Venkateshmurthy NS, Mohan S, Prabhakaran D. Hypertension in Low- and Middle-Income Countries. Circ Res. 2021;128(7):808-26.
7. Yan X, Mudiganti S, Husby H, Hudnut A, Gbotoe M, Jones JB. Medication non-adherence and therapeutic inertia independently contribute to poor disease control for cardiometabolic diseases. Sci Rep. 2022;12(1):18936.
8. Azharuddin M, Adil M, Sharma M, Gyawali B. A systematic review and meta-analysis of non-adherence to anti-diabetic medication: Evidence from low- and middle-income countries. Int J Clin Pract. 2021;75(11):e14717.
9. Morrison F, Shubina M, Turchin A. Encounter frequency and serum glucose level, blood pressure, and cholesterol level control in patients with diabetes mellitus. Arch Intern Med. 2011;171(17):1542-50.

10. Turchin A, Goldberg SI, Shubina M, Einbinder JS, Conlin PR. Encounter frequency and blood pressure in hypertensive patients with diabetes mellitus. Hypertension. 2010;56(1):68-74.
11. Jackson C, Ball L. Continuity of care: Vital, but how do we measure and promote it? Aust J Gen Pract. 2018;47(10):662-4.
12. Parchman ML, Pugh JA, Romero RL, Bowers KW. Competing demands or clinical inertia: the case of elevated glycosylated hemoglobin. Ann Fam Med. 2007;5(3):196-201.
13. Khunti S, Khunti K, Seidu S. Therapeutic inertia in type 2 diabetes: prevalence, causes, consequences and methods to overcome inertia. Ther Adv Endocrinol Metab. 2019;10:2042018819844694.
14. Willock RJ, Miller JB, Mohyi M, Abuzaanona A, Muminovic M, Levy PD. Therapeutic Inertia and Treatment Intensification. Curr Hypertens Rep. 2018;20(1):4.
15. Lall D, Engel N, Devadasan N, Horstman K, Criel B. Models of care for chronic conditions in low/middle-income countries: a 'best fit' framework synthesis. BMJ Glob Health. 2018;3(6):e001077.
16. Eaddy MT, Cook CL, O'Day K, Burch SP, Cantrell CR. How patient cost-sharing trends affect adherence and outcomes: a literature review. P T. 2012;37(1):45-55.
17. Morgan SG, Lee A. Cost-related non-adherence to prescribed medicines among older adults: a cross-sectional analysis of a survey in 11 developed countries. BMJ Open. 2017;7(1):e014287.
18. Macquart de Terline D, Kane A, Kramoh KE, Ali Toure I, Mipinda JB, Diop IB, et al. Factors associated with poor adherence to medication among hypertensive patients in twelve low and middle income Sub-Saharan countries. PLoS One. 2019;14(7):e0219266.
19. Hsu J, Price M, Huang J, Brand R, Fung V, Hui R, et al. Unintended consequences of caps on Medicare drug benefits. N Engl J Med. 2006;354(22):2349-59.
20. Barrera L, Oviedo D, Silva A, Tovar D, Méndez F. Continuity of Care and the Control of High Blood Pressure at Colombian Primary Care Services. Inquiry. 2021;58:469580211047043.
21. Chan KS, Wan EY, Chin WY, Cheng WH, Ho MK, Yu EY, et al. Effects of continuity of care on health outcomes among patients with diabetes mellitus and/or hypertension: a systematic review. BMC Fam Pract. 2021;22(1):145.
22. Xu W, Goldberg SI, Shubina M, Turchin A. Optimal systolic blood pressure target, time to intensification, and time to follow-up in treatment of hypertension: population based retrospective cohort study. BMJ. 2015;350:h158.
23. Pantalone KM, Wells BJ, Chagin KM, Ejzykowicz F, Yu C, Milinovich A, et al. Intensification of Diabetes Therapy and Time Until A1C Goal Attainment Among Patients With Newly Diagnosed Type 2 Diabetes Who Fail Metformin Monotherapy Within a Large Integrated Health System. Diabetes Care. 2016;39(9):1527-34.
24. Desai U, Kirson NY, Kim J, Khunti K, King S, Trieschman E, et al. Time to Treatment Intensification After Monotherapy Failure and Its Association With Subsequent Glycemic Control Among 93,515 Patients With Type 2 Diabetes. Diabetes Care. 2018;41(10):2096-104.
25. Arnold RJG, Yang S, Gold EJ, Farahbakhshian S, Sheehan JJ. Assessment of the relationship between diabetes treatment intensification and quality measure performance using electronic medical records. PLoS One. 2018;13(6):e0199011.
26. Maddox TM, Ross C, Tavel HM, Lyons EE, Tillquist M, Ho PM, et al. Blood pressure trajectories and associations with treatment intensification, medication adherence, and outcomes among newly diagnosed coronary artery disease patients. Circ Cardiovasc Qual Outcomes. 2010;3(4):347-57.
27. Paul SK, Klein K, Thorsted BL, Wolden ML, Khunti K. Delay in treatment intensification increases the risks of cardiovascular events in patients with type 2 diabetes. Cardiovasc Diabetol. 2015;14:100.
28. Goh LH, Siah CJR, Tam WWS, Tai ES, Young DYL. Effectiveness of the chronic care model for adults with type 2 diabetes in primary care: a systematic review and meta-analysis. Syst Rev. 2022;11(1):273.
29. Flood D, Hane J, Dunn M, Brown SJ, Wagenaar BH, Rogers EA, et al. Health system interventions for adults with type 2 diabetes in low- and middle-income countries: A systematic review and meta-analysis. PLoS Med. 2020;17(11):e1003434.
30. Guwatudde D, Delobelle P, Absetz P, Van JO, Mayega RW, Kasujja FX, et al. Prevention and management of type 2 diabetes mellitus in Uganda and South Africa: Findings from the SMART2D pragmatic implementation trial. PLOS Global Public Health. 2022;2(5):e0000425.

31. O'Toole TP, Buckel L, Bourgault C, Blumen J, Redihan SG, Jiang L, et al. Applying the chronic care model to homeless veterans: effect of a population approach to primary care on utilization and clinical outcomes. Am J Public Health. 2010;100(12):2493-9.
32. Barceló A, Cafiero E, de Boer M, Mesa AE, Lopez MG, Jiménez RA, et al. Using collaborative learning to improve diabetes care and outcomes: The VIDA project. Prim Care Diabetes. 2010;4(3):145-53.
33. Noda M, Hayashino Y, Yamazaki K, Suzuki H, Goto A, Kato M, et al. A cluster-randomized trial of the effectiveness of a triple-faceted intervention promoting adherence to primary care physician visits by diabetes patients. Sci Rep. 2020;10(1):2842.
34. Green LA, Wyszewianski L, Lowery JC, Kowalski CP, Krein SL. An observational study of the effectiveness of practice guideline implementation strategies examined according to physicians' cognitive styles. Implementation Science. 2007;2(1):41.
35. Reach G, Pechtner V, Gentilella R, Corcos A, Ceriello A. Clinical inertia and its impact on treatment intensification in people with type 2 diabetes mellitus. Diabetes Metab. 2017;43(6):501-11.
36. Brettler JW, Arcila GPG, Aumala T, Best A, Campbell NR, Cyr S, et al. Drivers and scorecards to improve hypertension control in primary care practice: Recommendations from the HEARTS in the Americas Innovation Group. Lancet Reg Health Am. 2022;9:None.
37. Russell-Jones D, Pouwer F, Khunti K. Identification of barriers to insulin therapy and approaches to overcoming them. Diabetes Obes Metab. 2018;20(3):488-96.
38. Baum HB, Cagliero E, Berry CA, Mencia WA, Stowell SA, Miller SC. Continuing improvement in type 2 diabetes care through performance-based evaluations. J Prim Care Community Health. 2014;5(2):107-11.
39. Viswanathan M, Golin CE, Jones CD, Ashok M, Blalock SJ, Wines RC, et al. Interventions to improve adherence to self-administered medications for chronic diseases in the United States: a systematic review. Ann Intern Med. 2012;157(11):785-95.
40. Mikhael EM, Hassali MA, Hussain SA. Effectiveness of Diabetes Self-Management Educational Programs For Type 2 Diabetes Mellitus Patients In Middle East Countries: A Systematic Review. Diabetes, Metabolic Syndrome and Obesity: Targets and Therapy. 2020;Volume 13:117-38.
41. Kim K, Choi JS, Choi E, Nieman CL, Joo JH, Lin FR, et al. Effects of Community-Based Health Worker Interventions to Improve Chronic Disease Management and Care Among Vulnerable Populations: A Systematic Review. Am J Public Health. 2016;106(4):e3-e28.
42. Brownstein JN, Chowdhury FM, Norris SL, Horsley T, Jack L, Jr., Zhang X, et al. Effectiveness of community health workers in the care of people with hypertension. Am J Prev Med. 2007;32(5):435-47.
43. Anand TN, Joseph LM, Geetha AV, Prabhakaran D, Jeemon P. Task sharing with non-physician health-care workers for management of blood pressure in low-income and middle-income countries: a systematic review and meta-analysis. The Lancet Global Health. 2019;7(6):e761-e71.
44. Palmas W, March D, Darakjy S, Findley SE, Teresi J, Carrasquillo O, et al. Community Health Worker Interventions to Improve Glycemic Control in People with Diabetes: A Systematic Review and Meta-Analysis. J Gen Intern Med. 2015;30(7):1004-12.
45. Maria JL, Anand TN, Dona B, Prinu J, Prabhakaran D, Jeemon P. Task-sharing interventions for improving control of diabetes in low-income and middle-income countries: a systematic review and meta-analysis. The Lancet Global Health. 2021;9(2):e170-e80.
46. Mbuthia GW, Magutah K, Pellowski J. Approaches and outcomes of community health worker's interventions for hypertension management and control in low-income and middle-income countries: systematic review. BMJ Open. 2022;12(4):e053455.
47. Franke MF, Kaigamba F, Socci AR, Hakizamungu M, Patel A, Bagiruwigize E, et al. Improved retention associated with community-based accompaniment for antiretroviral therapy delivery in rural Rwanda. Clin Infect Dis. 2013;56(9):1319-26.
48. Igumbor JO, Scheepers E, Ebrahim R, Jason A, Grimwood A. An evaluation of the impact of a community-based adherence support programme on ART outcomes in selected government HIV treatment sites in South Africa. AIDS Care. 2011;23(2):231-6.

49. Wroe EB, Nhlema B, Dunbar EL, Kulinkina AV, Kachimanga C, Aron M, et al. A household-based community health worker programme for non-communicable disease, malnutrition, tuberculosis, HIV and maternal health: a stepped-wedge cluster randomised controlled trial in Neno District, Malawi. BMJ Glob Health. 2021;6(9).
50. Jack HE, Arabadjis SD, Sun L, Sullivan EE, Phillips RS. Impact of Community Health Workers on Use of Healthcare Services in the United States: A Systematic Review. J Gen Intern Med. 2017;32(3):325-44.
51. Newman PM, Franke MF, Arrieta J, Carrasco H, Elliott P, Flores H, et al. Community health workers improve disease control and medication adherence among patients with diabetes and/or hypertension in Chiapas, Mexico: an observational stepped-wedge study. BMJ Global Health. 2018;3(1):e000566.
52. Rahul A, Chintha S, Anish TS, Prajitha KC, Indu PS. Effectiveness of a Non-pharmacological Intervention to Control Diabetes Mellitus in a Primary Care Setting in Kerala: A Cluster-Randomized Controlled Trial. Front Public Health. 2021;9:747065.
53. Njie GJ, Finnie RK, Acharya SD, Jacob V, Proia KK, Hopkins DP, et al. Reducing Medication Costs to Prevent Cardiovascular Disease: A Community Guide Systematic Review. Prev Chronic Dis. 2015;12:E208.
