# Supplementary material for: A retrospective cohort study of a community-based primary care program’s effects on pharmacotherapy quality in low-income Peruvians with type 2 diabetes and hypertension
Source: PLOS Glob Public Health. 2024 Aug 22;4(8):e0003512. doi: 10.1371/journal.pgph.0003512 (PMC11341050; doi:10.1371/journal.pgph.0003512)
Supplement: S1 File — (PDF) [file pgph.0003512.s002.pdf]

Program components were the Chronic Care Model (CCM), community health workers (CHWs), and low-cost access to care (free visits and medications).

CHRONIC CARE MODEL (CCM)

The program had four of six CCM elements, excluding the health system and clinical information systems (1):

- Delivery System Design, including, during the home care period, CHW home visits and remote physician treatment decisions.
- Self-Management
- Decision Support
- Community Resources.

The CCM elements of Health Care Organization and Clinical Information Systems were not included.

Delivery system design. Two models of care were employed sequentially, the home period for 10 months, then the clinic period for 17 months.

During the home care period, patients first had encounters with the physician during which medical histories were obtained, physical examinations were performed, medication treatment protocols were discussed (to which patients verbally consented), and information on current medications and their beneficial and adverse effects were discussed. Patients understood they would receive contemporaneous risk/benefit information about (and could decline) any medications later added based on the treatment protocol. Medication information is found at the DSME curriculum on the Siempre Salud Wiki. We modeled this approach to consent on studies of self-titration of hypoglycemic (1) and antihypertensive (2) medications in patients with diabetes and hypertension.

Thereafter, CHWs made weekly home visits during which they monitored (blood pressure and glucose); tracked behaviors (pill counts and diet recall); educated patients (using American Association of Diabetic Educators (AADE) National Standard 6 curriculum) (2); assisted patients with goal setting and completion; delivered medications and information about their schedules, indications, and side-effects; and documented visits using structured encounter forms. See home visit forms at Siempre Salud Wiki. They responded to clinical alerts and referred patients to emergency care in the event of severe hypoglycemia, emergent hypertension, or worrisome symptoms, e.g., chest pain or neurological symptoms. CHWs participated in monthly chart reviews during which they entered encounter data in Microsoft Excel spreadsheets and filled prescriptions under physician supervision. Physician treatment decisions were made remotely during physician treatment conferences attended by the physician and CHWs. Patients were not present during conferences. Occasionally, in-person visits were necessary before treatment. In those cases, the physician made visits to patients' homes.

During the clinic period, patients had all encounters with the same physician in a centralized clinic. Clinic sessions devoted to chronic diseases were held during six consecutive days of each month. Clinic hours were 5 AM to 1 PM on Monday and Thursday, 10 AM to 6 PM on Tuesday and Friday, and 4 PM to midnight on Wednesday and Saturday, i.e., each three-day period offered visits between 5 AM and midnight. All visits were unscheduled. Patients were seen in the order they appeared in the clinic. The CHWs acted as clinic assistants and obtained clinical measures (blood pressure, weight, and glucose), counted pills, and obtained a diet recall. The physician was responsible for treatment decisions, patient education including discussion of medication benefits and adverse effects (including written information found at DSME curriculum on the Siempre Salud Wiki) and discussing patients' goals, attending to other primary care needs, and documentation of encounters. See clinic visit forms at Siempre Salud Wiki. CHWs in the clinic filled prescriptions under physician supervision.

Self-management. The diabetes self-management education (DSME) curriculum was based on the first seven of nine content areas of Standard 6 (curriculum) of the AADE national DSME standards (2): 1) disease process and treatment, 2) nutritional management, 3) physical activity, 4) medication use, 5) glucose monitoring, 6) acute complications, 7) chronic complications, 8) strategies to address psychosocial issues, 9) strategies to address health and behavior change.

DSME was provided by CHWs during the home care period and by the physician during the clinic period. A physician trained CHWs in the AADE National Standard 6 curriculum, content areas 1-7 (2).

DSME materials used for CHW training and patient care are from

- American Diabetes Association (ADA) (https://diabetes.org/),
- National Institute of Diabetes and Digestive and Kidney Diseases (NIDDK) (https://www.niddk.nih.gov/health-information/informacion-de-la-salud/diabetes),
- Mayo Cinic (https://www.mayoclinic.org/es/diseases-conditions), and
- Drugs.com (https://www.drugs.com/).

See DSME curriculum at the Siempre Salud Wiki.

We do not formally train in AADE National Standard 6 content areas 8 and 9 (psychosocial issues and behavior change) [2]. At each visit, DSME topics discussed are itemized. Topics are self-selected by patients in weekly home or monthly clinic visits according to AADE statement that “needs of individuals with …. diabetes will determine which of the content areas … are to be provided” (2).

We follow ADA/AADE National Standard 9 (monitoring of behavior change) (2) but only with respect to the three of the seven self-care behaviors (3): 1) healthy eating (diet recall and carbohydrate counting and evaluation each visit), 2) glucose monitoring, and 3) taking medication (pill counting each visit). We do not monitor 1) physical activity, 2), problem-solving, 6) health coping, and 7) reducing risks. We do not measure patient diabetes self-management knowledge.

The curriculum for hypertension self-management education is based on materials from websites of the

- American Heart Association (Heart.org)
- Department of Health and Human Services (HHS) (millionhearts.hhs.gov)
- National Heart Lung Blood Institute (https://www.nhlbi.nih.gov/education/high-blood-pressure).

See the hypertension self-management curriculum at the Siempre Salud Wiki.

Decision support. Guidelines were reviewed, itemized, reconciled, consolidated, and adapted to our low-resource setting, then converted to condensed treatment protocols. Guidelines reviewed are from:

- American Diabetes Association (ADA) (4, 5);
- American Association of Diabetic Educators (AADE) (2, 3);
- American Heart Association (AHA) (6);
- Joint National Committee (JNC) on Prevention, Detection, Evaluation, and Treatment of High Blood Pressure (7);
- National Cholesterol Education Program (NCEP) (8);
- World Health Organization (WHO) (9, 10)

The final Siempre Salud standards of care were those items adopted based on their availability, feasibility, and affordability in our low-resource setting. See the Siempre Salud standards of primary care of type 2 diabetes and prevention of cardiovascular disease (CVD), including hypertension management, at the Siempre Salud Wiki. We did not adopt any of the standards of pharmacotherapy of dyslipidemia because lipid testing and statin drugs were unavailable due to cost. The WHO does not recommend statin treatment if it would compromise the availability of antihypertensive and hypoglycemic agents (10).

To simplify decision-making, the Siempre Salud standards (as adopted) were converted to simple medication treatment protocols which also conformed to "Table 4. Oral Antihypertensive Drugs" and "Figure. Antihypertensive Treatment Algorithm" from the seventh JNC report (7). See pharmacotherapy protocols at Siempre Salud Wiki. The burden on decision-making was further lessened by limiting the formulary to one agent from each of seven classes of medications:

1. Metformin (biguanide)
2. Glibenclamide (sulfonylurea)
3. Hydrochlorothiazide (thiazide diuretic)
4. Enalapril (ACEi),
5. Amlodipine (calcium-channel blocker (CCB))
6. Atenolol (beta-adrenergic blocker (BB))
7. Low-dose aspirin.

These medications also appear in the WHO Package of Essential Noncommunicable Disease Interventions (10) for low-resource settings.

Community resources. The community participating in the program consists of three neighborhoods which are organized as associations. Associations elect officers (President, Vice-President, etc.) who convene regular meetings of the Asamblea General (General Assembly) which is open to all community members. Community mobilization to participate in and support the program was achieved through meetings with and presentations to the General Assembly and by residents who made presentations, in a variety of settings (churches, schools, street corners, and parks) on premature adult mortality prevention. Other community resources included volunteer surveyors; other community spaces; and leadership support. All CHWs lived within the neighborhoods they served.

PROGRAM STAFF

Community Health Workers (CHWs):

- Five female CHWs participated in the program.
- CHWs lived in the communities they served.
- All had completed formal secondary education. None had post-secondary education.
- Each followed caseloads of 25-35 patients.
- Prior to becoming CHWs, they all had prior experience as volunteers with Asociación Siempre Salud. They had been door-to-door household surveyors; presenters of a community seminar, "Prevención de Muertes Prematuras de Adultos en el Perú" (Prevention of Premature Adult Mortality in Peru); and assistants during community-wide diabetes and hypertension screening.
- Remuneration. CHWs were paid Peru's prevailing minimum wage converted to an hourly rate.
- Training (didactic) included included 52 hours of lectures, group interactive sessions, role-playing, and practice in diabetes self-management education (DSME), clinical skills (height, weight, blood pressure, glucose monitoring, diet recall, carbohydrate counting, and pill-counting), individualized goal setting, and documentation and record-keeping. The lecture series consisted of items 1-7 of the AADE National Standards, standard 6, content areas 1-7 (2).
- Training (practical and ongoing). The CHWs participate in a series of supervised visits before visiting patients independently. The monthly physician treatment conference is also a school where CHWs present their patients (of which each has 25-30) and receive guidance and continual learning.
- Supervision. Patients are regularly contacted for their evaluations of their home visits.
- CHW knowledge tested using Diabetes Knowledge Questionnaire DKQ (11). Knowledge was re-assessed until a score of 100% was achieved. See DKQ at Siempre Salud Wiki.

Physician

- John E. Deaver, MD is the program's only physician. See curriculum vitae at Siempre Salud Wiki.

- Graduate of University of Iowa (BS and MD), University of Texas Health Science Center at Houston School of Public Health (MS, Epidemiology), New York-Presbyterian Hospital, Columbia University Medical Center (residency, obstetrics and gynecology), and Albert Einstein College of Medicine, Bronx, New York, USA (fellowship, maternal-fetal medicine).
- He is the former Medical Director of Medisys Women's Health Center Jamaica Hospital (Queens, New York) where he was also the Director of the Diabetes in Pregnancy Program (DiPP).
- Founder and Executive Director of Asociación Siempre Salud, a non-profit organization in Peru.
- Certified to practice medicine in Perú by Colégio Médico del Perú (Numero de Colegiatura 076375)
- Diplomate of the American Board of Obstetrics and Gynecology.

REFERENCES

(1) Bodenheimer T, Wagner EH, Grumbach K. Improving primary care for patients with chronic illness. JAMA. 2002;288(14):1775-9.

(2) Funnell, M., Brown, T., Childs, B., Haas, L., Hosey, G., Jensen, B., Maryniuk, M., Peyrot, M., Piette, J., Reader, D., Siminerio, L., Weinger, K., & Weiss, M. (2007). National standards for diabetes self-management education. *Diabetes Care*, *30*(6), 1630–1637. https://doi.org/10.2337/DC07-9923

(3) American Association of Diabetes Educators. (2011). *AADE 7 Self-Care Behaviors American Association of Diabetes Educators (AADE) Position Statement*. https://www.diabeteseducator.org/docs/default-source/practice/practice-resources/position-statements/aade7-self-care-behaviors-position-statement.pdf?sfvrsn=6

(4) American Diabetes Association. (2011). Standards of Medical Care in Diabetes—2011. *Diabetes Care*, *34*(Supplement 1), S11–S61. https://doi.org/10.2337/DC11-S011

(5) American Diabetes Association. (2008). Nutrition Recommendations and Interventions for Diabetes. *Diabetes Care*, *31*(Supplement 1), S61–S78. https://doi.org/10.2337/DC08-S061

(6) Pearson TA, Blair SN, Daniels SR, Eckel RH, Fair JM, Fortmann SP, et al. AHA Guidelines for Primary Prevention of Cardiovascular Disease and Stroke: 2002 Update: Consensus Panel Guide to Comprehensive Risk Reduction for Adult Patients Without Coronary or Other Atherosclerotic Vascular Diseases. American Heart Association Science Advisory and Coordinating Committee. Circulation. 2002;106(3):388-91.

(7) Chobanian AV, Bakris GL, Black HR, Cushman WC, Green LA, Izzo JL, Jr., et al. The Seventh Report of the Joint National Committee on Prevention, Detection, Evaluation, and Treatment of High Blood Pressure: the JNC 7 report. JAMA. 2003;289(19):2560-72.

(8) Executive Summary of The Third Report of The National Cholesterol Education Program (NCEP) Expert Panel on Detection, Evaluation, And Treatment of High Blood Cholesterol In Adults (Adult Treatment Panel III). JAMA. 2001;285(19):2486-97.
.

(9) World Health Organization. Prevention of cardiovascular disease. Guidelines for the assessment and management of total cardiovascular risk. Geneva, Switzerland: World Health Organization; 2007.

(10) World Health Organization. Package of Essential Noncommunicable (PEN) Disease Interventions for Primary Health Care in Low-Resource Settings. Geneva, Switzerland: World Health Organization; 2010. 1-66 p.

(11) Garcia, A., Villagomez, E., Brown, S., Kouzekanani, K., & Hanis, C. (2001). The Starr County Diabetes Education Study: development of the Spanish-language diabetes knowledge questionnaire. *Diabetes Care*, *24*(1), 16–21. https://doi.org/10.2337/DIACARE.24.1.16

(12) Nagrebetsky A, Larsen M, Craven A, Turner J, McRobert N, Murray E, et al. Stepwise Self-Titration of Oral Glucose-Lowering Medication Using a Mobile Telephone-Based Telehealth Platform in Type 2 Diabetes: A Feasibility Trial in Primary Care. J Diabetes Sci Technol. 2013;7(1):123-34.

(13) McManus RJ, Mant J, Haque MS, Bray EP, Bryan S, Greenfield SM, et al. Effect of Self-monitoring and Medication Self-titration on Systolic Blood Pressure in Hypertensive Patients at High Risk of Cardiovascular Disease. JAMA. 2014;312(8):799.
