# Supplementary material for: A retrospective cohort study of a community-based primary care program’s effects on pharmacotherapy quality in low-income Peruvians with type 2 diabetes and hypertension
Source: PLOS Glob Public Health. 2024 Aug 22;4(8):e0003512. doi: 10.1371/journal.pgph.0003512 (PMC11341050; doi:10.1371/journal.pgph.0003512)
Supplement: S2 File — (PDF) [file pgph.0003512.s003.pdf]

STROBE Statement Checklist of items that should be included in reports of ***cohort studies***

A prospective cohort study of a community-based primary care program's effects on pharmacotherapy quality in low-income Peruvians with type 2 diabetes and hypertension (as submitted).

	Item No	Recommendation	Status
**Title and abstract**	1	(*a*) Indicate the study's design with a commonly used term in the title or the abstract	Completed. See Title and Abstact
		(*b*) Provide in the abstract an informative and balanced summary of what was done and what was found	Completed. See Abstract.
**Introduction**			
Background/rationale	2	Explain the scientific background and rationale for the investigation being reported	Completed. See Introduction
Objectives	3	State specific objectives, including any prespecified hypotheses	Completed. See Introduction (last paragraph).
**Methods**			
Study design	4	Present key elements of study design early in the paper	Completed. See Introduction and Methods.
Setting	5	Describe the setting, locations, and relevant dates, including periods of recruitment, exposure, follow-up, and data collection	Completed. See Methods and Figure 1
Participants	6	(*a*) Give the eligibility criteria, and the sources and methods of selection of participants. Describe methods of follow-up	Completed. See Methods.
		(*b*) For matched studies, give matching criteria and number of exposed and unexposed	Completed. See Methods, Data analysis (only the study of 'program' effects was a matched analysis).
Variables	7	Clearly define all outcomes, exposures, predictors, potential confounders, and effect modifiers. Give diagnostic criteria, if applicable	Completed. See Methods, Exposure and Outcome Measures. See Methods, Statistical Analysis for confounder and effect modifiers.
Data sources/ measurement	8*	For each variable of interest, give sources of data and details of methods of assessment (measurement). Describe comparability of assessment methods if there is more than	Completed. See Methods, Exposures, Outcomes, Covariates.

		one group	
Bias	9	Describe any efforts to address potential sources of bias	Completed. See Methods, Statistical analysis; and Discussion, Limitations.
Study size	10	Explain how the study size was arrived at	Completed. See Methods, Statistical analysis (last sentence). See post hoc power calculation for analysis of care model effects in Results.
Quantitative variables	11	Explain how quantitative variables were handled in the analyses. If applicable, describe which groupings were chosen and why	Completed. See Methods, Exposure, Outcome measures, and Covariates.
Statistical methods	12	(*a*) Describe all statistical methods, including those used to control for confounding	Completed. See Methods, Statistical Analysis.
		(*b*) Describe any methods used to examine subgroups and interactions	Not applicable. See Methods, Statistical Analysis (effect modification)
		(*c*) Explain how missing data were addressed	See Methods, Data analysis (tirst sentence).
		(*d*) If applicable, explain how loss to follow-up was addressed	Completed. See Methods, Data analysis (paragraphs 2-4). All patients included. No loss ot follow-up. Retention in care studied as exposure (program effects) or independent variable (care period effects).
		(*e*) Describe any sensitivity analyses	Not applicable.
**Results**			
Participants	13*	(a) Report numbers of individuals at each stage of study—eg numbers potentially eligible, examined for eligibility, confirmed eligible, included in the study, completing follow-up, and analysed	Completed. See Methods and Figure 1.
		(b) Give reasons for non-participation at each stage	See Methods and Figure 1.
		(c) Consider use of a flow diagram	See Figure 1.
Descriptive data	14*	(a) Give characteristics of study participants (eg demographic, clinical, social) and information on exposures and potential confounders	Completed. See Table 1. .
		(b) Indicate number of participants with missing data for each variable of interest	Completed. See missing values in footnotes of tables 1-3.
		(c) Summarise follow-up time	Completed. See results, retention in care (descriptive)

		(eg, average and total amount)	for median follow-up by care periods.
Outcome data	15*	Report numbers of outcome events or summary measures over time	Completed. See Table 2.
Main results	16	(*a*) Give unadjusted estimates and, if applicable, confounder-adjusted estimates and their precision (eg, 95% confidence interval). Make clear which confounders were adjusted for and why they were included	Completed. See Tables 2 for unadjusted estimates of all exposure. See Table 3 for adjusted estimates of care period exposure.
		(*b*) Report category boundaries when continuous variables were categorized	Completed but not included in manuscript.
		(*c*) If relevant, consider translating estimates of relative risk into absolute risk for a meaningful time period	Completed. See Table 2 for risks and relative risks. See Table 3 for odds ratios and predicted probabilities.
Other analyses	17	Report other analyses done—eg analyses of subgroups and interactions, and sensitivity analyses	Not applicable.
**Discussion**			
Key results	18	Summarise key results with reference to study objectives	Completed. See Discussion.
Limitations	19	Discuss limitations of the study, taking into account sources of potential bias or imprecision. Discuss both direction and magnitude of any potential bias	Completed. See Discussion, Limitations.
Interpretation	20	Give a cautious overall interpretation of results considering objectives, limitations, multiplicity of analyses, results from similar studies, and other relevant evidence	Completed. See Discussion.
Generalisability	21	Discuss the generalisability (external validity) of the study results	Completed. See Discussion, Limitations
**Other information**			
Funding	22	Give the source of funding and the role of the funders for the present study and, if applicable, for the original study on which the present article is based	Completed. See funding source following the manuscript’s references.

*Give information separately for exposed and unexposed groups.

Note: An Explanation and Elaboration article discusses each checklist item and gives methodological background and published examples of transparent reporting. The STROBE checklist is best used in conjunction with this article (freely available on the Web sites of PLoS Medicine at http://www.plosmedicine.org/, Annals of Internal Medicine at http://www.annals.org/, and Epidemiology at http://www.epidem.com/). Information on the STROBE Initiative is available at http://www.strobe-statement.org.
