# Supplementary material for: A retrospective cohort study of a community-based primary care program’s effects on pharmacotherapy quality in low-income Peruvians with type 2 diabetes and hypertension
Source: PLOS Glob Public Health. 2024 Aug 22;4(8):e0003512. doi: 10.1371/journal.pgph.0003512 (PMC11341050; doi:10.1371/journal.pgph.0003512)
Supplement: S1 Table — (PDF) [file pgph.0003512.s004.pdf]

Supplemental information

S1 Table. Siempre Salud standards of care for type 2 diabetes and hypertension

S1 Table, Part 1. Standards of Type 2 Diabetes Care (as adopted)

Category	ADA Standards of Medical Care 2011 [1]	Adopted by Siempre Salud	footnote #
Diagnosis of diabetes	Fasting glucose 126 mg/dl (7 mmol/L) or greater, repeat for confirmation	Yes	
Initial evaluation - hx	Medical history	Yes	
Initial evaluation – pe	BMI	Yes	
Initial evaluation – pe	BP	Yes	
Initial evaluation – pe	Thyroid palpation	Yes	
Initial evaluation – pe	Skin exam	Yes	
Initial eval – referrals	Diabetes Self-Management Education (DSME) National Standards, Standard 6 (curriculum) [2] “needs of individuals with …. diabetes will determine which of the content areas … are to be provided.” Content areas:<br>1) disease process and treatment,<br>2) nutritional management,<br>3) physical activity,<br>4) medication use,<br>5) glucose monitoring,<br>6) acute complications,<br>7) chronic complications,<br>8) strategies to address psychosocial issues,<br>9) strategies to address health and behavior change.	Yes	4
Initial eval – referrals	Dental exam	Yes	
Glycemic control	Self-monitoring blood glucose (>= 3 x daily for insulin; no recommended frequency for non-insulin therapy)	Yes	12
Glycemic control	Glucose monitor teaching	Yes	13
Glycemic control	Pre- and post-prandial values useful	Yes	
Glycemic control	Adjust diet, activity, pharmacotherapy to achieve targets	Yes	
Glycemic control	Pre-prandial glucose <130 mg/dl (<7.2 mmol/L), postprandial <180 mg/dl (<10.0 mmol/L)	Yes	
Pharmacotherapy (hypoglycemics)	MNT/exercise, then metformin, then other tier 1: sulfonylurea or insulin. TZDs and GLP-1 agonists are tier 2.	Yes	7, 14
DSME	DSME by National Standards at diagnosis and periodically	Yes	4
DSME	DSME measured and monitored	Yes	4
Medical nutrition therapy [3]	Individualized	Yes	
Medical nutrition therapy [3]	Monitor carbohydrates by carb counting, choices, or experience-based estimation	Yes	
Medical nutrition therapy [3]	Use glycemic index and glycemic load	Yes	
Medical nutrition therapy [3]	Saturated fat <7% of calories	Yes	
Medical nutrition therapy [3]	Minimize trans fat intake	Yes	
Medical nutrition therapy [3]	Meet RDA for all micronutrients	Yes	
Weight loss	Physical activity	Yes	
Physical activity	150 min/week of moderate intensity aerobic physical activity	Yes	
Hypoglycemia	15 grams of glucose or carbohydrate, repeat in 15 minutes as needed. Eat a snack or meal to prevent recurrence.	Yes	

Supplemental information

S1 Table, Part 1. Standards of Type 2 Diabetes Care (as adopted)

Category	ADA Standards of Medical Care 2011 [1]	Adopted by Siempre Salud	footnote #
CVD – blood pressure	Blood pressure at every visit	Yes	
CVD – blood pressure	SBP >130, DBP >80, confirmed on separate day, is diagnostic of hypertension for treatment purposes in diabetes	Yes	
CVD – blood pressure	Target is SBP <130, DBP <80	Yes	
CVD – blood pressure	Brief (3 m) trial of lifestyle change 130-139/80-89, then pharmacologic	Yes	
CVD – blood pressure	140/90 or higher, immediate pharmacotherapy	Yes	
CVD – blood pressure	DASH diet	Yes	
CVD – blood pressure	Use ACEi or ARB as first-line Rx	Yes	
CVD – blood pressure	Add thiazide or loop diuretic as 2nd drug	Yes	
CVD – blood pressure	Add other classes of hypertension medications as needed for control	Yes	10
CVD – blood pressure	If refractory to max doses of 3 or more Rx, then evaluate for secondary hypertension	Yes	11
CVD – lipids	Statin should be added, regardless of lipid profile for 1) overt CVD, or 2) >40 years old and one or more other CVD risk factors (essentially those older patients with hypertension or obesity in our population)	Yes	5
CVD – lipids	Lifestyle interventions (diet, activity, weight loss, smoking cessation	Yes	
CVD – lipids	Diet low in saturated and trans fats and cholesterol and high in omega-3-fatty acids, viscous fiber, and plant stanols/sterols	Yes	
CVD – lipids	Glycemic control to lower lipids especially triglycerides	Yes	
CVD risk - Anti-platelets	Secondary prevention: low-dose aspirin in all patient with CVD history	Yes	
CVD risk - Anti-platelets	Primary prevention: low-dose aspriin when 10-yr ASCVD risk >10% (men/women >50/60 with >=1 additional CVD risk factor); use clinical judgement when risk is 5-10%	Yes	8
CVD risk - Anti-platelets	Don't use low-dose aspirin when 10-yr ASCVD risk <5%	Yes	
CVD risk - Anti-platelets	Clopidogrel substituted for aspirin when aspirin-allergic	Yes	
CVD risk - Anti-platelets	Clopidogrel + aspirin for one year in acute coronary syndrome	Yes	
CVD risk - Smoking cessation	Urge all smokers to quit	Yes	9
CVD risk - Smoking cessation	Cessation counseling and treatment	Yes	9
CHD screening and treatment	Screening for CAD is not recommended	Yes	
CHD screening and treatment	Knnown CVD: ACE + aspirin + statin	Yes	
CHD screening and treatment	Prior MI: beta-blocker for two years	Yes	
CHD screening and treatment	Avoid TZD in patients with CHF	Yes	
Nephropathy screening and treatment	Optimize glycemic control	Yes	
Nephropathy screening and treatment	Optimize blood pressure control	Yes	

Supplemental information

S1 Table, Part 1. Standards of Type 2 Diabetes Care (as adopted)

Category	ADA Standards of Medical Care 2011 [1]	Adopted by Siempre Salud	footnote #
Retinopathy screening and treatment	Optimize glycemic control	Yes	
Retinopathy screening and treatment	Optimize blood pressure control	Yes	
Neuropathy screening and treatment	Optimize glycemic control	Yes	
Community screening	Not recommended because people with positive tests may not seek nor have access to follow-up care	No	1
Initial evaluation – pe	Comprehensive foot exam	No	
Initial evaluation – lab	hemoglobin A1c	No	2
Initial evaluation – lab	Lipid profile	No	2
Initial evaluation – lab	Liver function tests	No	2
Initial evaluation – lab	Urine albumin-to-creatinine	No	2
Initial evaluation – lab	Creatinine	No	2
Initial evaluation – lab	TSH (female > 50 or hyperlipidemia)	No	2
Initial eval – referrals	Dilated fundoscopic exam	No	3
Initial eval – referrals	Registered dietician	No	3
Glycemic control	hemoglobin A1c 2 times yearly, q 3 m unmet goals or therapy changes	No	2
Glycemic control	POC hemoglobin A1c appropriate for monitoring (not dx)	No	5
Glycemic control	hemoglobin A1c goal <7%	No	6
DSME	Address psychosocial issues	No	4
Medical nutrition therapy [3]	Registered dietician	No	3
Weight loss	Weight loss is recommended for all overweight or obese diabetics	No	
Weight loss	Low-fat, low carbohydrate (<130 gm/d of carbs) or Mediterranean effective in short-term	No	
Weight loss	Behavior modification	No	
Physical activity	Resistance training 3 times per week	No	
CVD – blood pressure	Monitor Cr and K on ACEi/ARB	No	2
CVD – lipids	Fasting lipid profile annually in most	No	2
CVD – lipids	Target LDL <100 mg/dl in those without overt CVD	No	6
CVD – lipids	Target LDL <70 mg/dl in those with overt CVD	No	6
CVD – lipids	Alternative target is LDL 30-40% below baseline	No	6
CVD – lipids	Target triglycerides <150 mg/dl desirable (but primary target is LDL)	No	6
CVD – lipids	Target HDL >40 mg/dl (men) or >50 mg/dl (women) (but primary target is LDL)	No	6
CVD – lipids	Other lipid-lowering drugs if target not reached with a statin (uncertain effect CVD outcomes or safety)	No	6
CVD – lipids	Severe hypertriglyceridemia: treat with niacin, fibric acid derivative, or fish oil)	No	6
CVD – lipids	Low HDL: most effective Rx is niacin	No	6
CHD screening and treatment	Metformin may be used in stable CHF with normal renal function	No	6

Supplemental information

S1 Table, Part 1. Standards of Type 2 Diabetes Care (as adopted)

Category	ADA Standards of Medical Care 2011 [1]	Adopted by Siempre Salud	footnote #
Nephropathy screening and treatment	Urine albumin-to-creatinine test annually	No	2
Nephropathy screening and treatment	Serum creatinine annually	No	2
Nephropathy screening and treatment	Treat albuminuria (micro or macro) with ACEi/ARB	No	6
Nephropathy screening and treatment	Reduction of protein intake in early-stage CKD	No	6
Nephropathy screening and treatment	ACEi/ARB use: monitor serum potassium and creatinine	No	2
Nephropathy screening and treatment	When eGFR <60 ml/min, evaluate and manage complications of CKD	No	6
Retinopathy screening and treatment	Dilated fundoscopic exams annually	No	3
Retinopathy screening and treatment	Refer patients with macular edema, severe NPDR, or any PDR to ophtalmologist	No	3
Retinopathy screening and treatment	Laser photocoagulation is indicated for macular edema, high-risk PDR, and some cases of severe NPDR	No	3
Neuropathy screening and treatment	All patients should be screened for distal symmetrical polyneuropathy (DPR) annually using simple clinical tests	No	
Neuropathy screening and treatment	Screen for signs and symptoms of autonomic neuropathy (resting tachycardia, exercise intolerance, orthostatic hypotension, constipation, gastroparesis (nausea, vomiting, weight loss, feeling full too soon, abdominal bloating or pain, heartburn), erective dysfunction, sudomotor dysfunction (sweating too much or too little), impaired neurovascular function, autonomic failure (epinephrine counter-regulation) in response to hypoglycemia)	No	3
Neuropathy screening and treatment	Medications for symptom relief	No	3
Neuropathy screening and treatment	Metoclopramide (gastroparesis) and phosphodiesterase inhibitors (erectile dysfunction)	No	3
Foot care	Comprehensive annual foot exam: inspection, pulses, loss of protective sensation, the latter with 10-gram monofilament + one of vibration, pinprick, ankle refex, or vibration sensation threshold.	No	
Foot care	Refer foot ulcer for multidisciplinary care	No	3
Foot care	Refer to foot specialist: smoker, loss of protective sensation and structural abnormality, or prior lower extremity ulcer for ongoing preventive care and life-long surveillance	No	3
Foot care	Assess for PAD: history of claudication, pedal pulses.	No	3
Foot care	Assess for PAD: ankle-brachial index (ABI)	No	3
Foot care	Refer patients with suspected PAD for further assessment and treatment of exercise, medications, and surgery.	No	3

S1 Table, Part 1. Standards of Type 2 Diabetes Care (footnotes and references)

Number	Footnote
1	Community (population-based) testing is justified because we offer care to all existing (diagnosis-aware prior to screening by Siempre Salud)) and newly-diagnosed diabetics (diagnosis-unaware prior to screening).
2	laboratory testing unavailable due to inadequate funds for point-of-care testing and lack of quality laboratories in the area
3	No specialist in the area
4	The qualification of 'certified diabetes educator' does not exist in our region. A physician trains CHWs in ADA/AADE National Standard 6, Curriculum, content areas 1-7 (diabetes process and treatment, nutrition, physical activity, medications, glucose monitoring, acute complications, chronic complications) [2]. DSME materials used for CHW training and patient care are from ADA, NIDDK, Mayo Cinic, and Drugs.com. See DSME curriculum at Siempre Salud Wiki.<br>DSME Curriculum<br>We do not formally train in ADA/AADE National Standard 6 content areas 8 and 9 (psychosocial issues and behavior change) [2] . At each visit we itemize DSME topics discussed. We follow ADA/AADE National Standard 9 (monitoring of behavior change)and track the following self-care behaviors [4]: 1) healthy eating (diet recall and carbohydrate counting and evaluation each visit), 2) glucose monitoring, and 3) taking medication (pill counting each visit). We do not monitor 1) physicial activity, 2), problem-solving, 6) health coping, and 7) reducing risks.<br>We do not measure patient self-management knowledge. We measure CHW knowledge by Diabetes Knowledge Questionnaire (DKQ) [5] and re-assess until each CHW achieves score of 100%. See DKQ at Siempre Salud Wiki.<br>Diabetes Knowledge Questionnaire (DKQ)
5	Limited by the high cost of statin medications.
6	Target is based on a test that is not performed
7	As a practical matter, insulin not available (cost of drug and supplies, lack of refrigeration, patient objection to self-injection)
8	We provide low-dose aspirin for 10-year CVD risk >=10%. We calculate CVD risk with Framingham 2008, the "simple" BMI-based version [6]. The BMI-based risk calculator is available as download from Framington Heart Study page [7]. We use this because our patients generally cannot obtain lipid testing.
9	Tobacco smoking is rare in the communities we serve. We have no smokers in our diabetic population.
10	For hypertension, our formulary has enalapril (ACEi), losartan (ARB), hydrocholorothiazide (diuretic), amlodipine (calcium-channel antagonist), and atenolol (beta-blocker)
11	referred to cardiologist
12	home visits: weekly fasting or postprandial glucose; clinic visits: monthly fasting glucose
13	SiSa trained CHWs who performed the tests for patients
14	Meformin start 500-850 mg daily, increase incrementally based on glucose values to maximum dose of 2550 mg. Glibenclamide start at 5 mg daily and increase incrementally as needed to maximum dose of 20 mg (divided dosing twice daily) [8].

Number Reference

1 American Diabetes Association. (2011). Standards of Medical Care in Diabetes—2011. *Diabetes Care* , *34* (Supplement 1), S11–S61. https://doi.org/10.2337/DC11-S011

2 Funnell, M., Brown, T., Childs, B., Haas, L., Hosey, G., Jensen, B., Maryniuk, M., Peyrot, M., Piette, J., Reader, D., Siminerio, L., Weinger, K., & Weiss, M. (2007). National standards for diabetes self-management education. *Diabetes Care* , *30* (6), 1630–1637. https://doi.org/10.2337/DC07-9923

S1 Table, Part 1. Standards of Type 2 Diabetes Care (footnotes and references)

3 American Diabetes Association. (2008). Nutrition Recommendations and Interventions for Diabetes. *Diabetes Care* , *31* (Supplement 1), S61–S78. https://doi.org/10.2337/DC08-S061

4 American Association of Diabetes Educators. (2011). *AADE 7™ Self-Care Behaviors American Association of Diabetes Educators (AADE) Position Statement* . https://www.diabeteseducator.org/docs/default-source/practice/practice-resources/position-statements/aade7-self-care-behaviors-position-statement.pdf?sfvrsn=6

5 Garcia A, Villagomez E, Brown S, Kouzekanani K, Hanis C. The Starr County Diabetes Education Study: development of the Spanish-language diabetes knowledge questionnaire. Diabetes Care [Internet]. 2001;24(1):16–21

6 D'Agostino, R. B., Vasan, R. S., Pencina, M. J., Wolf, P. A., Cobain, M., Massaro, J. M., & Kannel, W. B. (2008). General cardiovascular risk profile for use in primary care: The Framingham heart study. *Circulation* , *117* (6), 743–753. https://doi.org/10.1161/CIRCULATIONAHA.107.699579

7 *Cardiovascular Disease (10-year risk)* | *Framingham Heart Study* . (n.d.). Retrieved March 12, 2022, from https://framinghamheartstudy.org/fhs-risk-functions/cardiovascular-disease-10-year-risk/

8 Nathan, D. M., Buse, J. B., Davidson, M. B., Ferrannini, E., Holman, R. R., Sherwin, R., & Zinman, B. (2009). Medical Management of Hyperglycemia in Type 2 Diabetes: A Consensus Algorithm for the Initiation and Adjustment of Therapy. *Diabetes Care* , *32* (1), 193–203. https://doi.org/10.2337/DC08-9025

Supporting information

S1 Table, Part 2. Standards of Cardiovascular Disease (CVD) Prevention (as adopted)

guideline	Recommendations	Reference	Adopted by Siempre Salud	Note
hypertension	In patients with diabetes, blood pressure should be measured at every routine visit. Patients found to have systolic blood pressure 130 mmHg or diastolic blood pressure 80 mmHg should have blood pressure confirmed on a separate day. Repeat systolic blood pressure >=130 mmHg or diastolic blood pressure >=80 mm Hg confirms a diagnosis of hypertension in diabetic patients. (C)	ADA 2011 (4)	yes	
hypertension	Use auscultatory method, patient seated upright in chair for at least five minutes, feet on the floor, arm positioned so that cuff is at heart level, appropriate sized cuff (cuff bladder covering 80% of arm circumference), two measurements should be made, SBP is the appearance of first sound, DBP is disappearnace of last sound, inform patient of BP results and goals.	JNC7 2003 (2)	yes	
hypertension	lifestyle and other cardiovascular risk factors	JNC7 2003 (2)	yes	
hypertension	identify causes of hypertension	JNC7 2003 (2)	yes	
hypertension	assess for presence of target organ damage and CVD	JNC7 2003 (2)	yes	
hypertension	BP in the contralateral arm	JNC7 2003 (2)	yes	
hypertension	calculation of BMI	JNC7 2003 (2)	yes	
hypertension	examination of optic fundi	JNC7 2003 (2)	yes	
hypertension	palpation of thyroid gland	JNC7 2003 (2)	yes	
hypertension	examination of heart and lungs	JNC7 2003 (2)	yes	
hypertension	examination of abdomen for enlarged kidneys, masses, abdominal aortic pulsations	JNC7 2003 (2)	yes	
hypertension	palpation of the lower extremities for edema and pulses	JNC7 2003 (2)	yes	
hypertension	neurological assessment	JNC7 2003 (2)	yes	
hypertension	auscultation for carotid, abdominal, and femoral bruits	JNC7 2003 (2)	yes	
hypertension	urinalysis	JNC7 2003 (2)	yes	
hypertension	glucose	JNC7 2003 (2)	yes	
hypertension	<140/90 or <130/80 in diabetes and chronic kidney disease	JNC7 2003 (2)	yes	

Supporting information

guideline	Recommendations	Reference	Adopted by Siempre Salud	Note
hypertension	Blood pressure goals: <140/90 mm Hg; <130/85 mm Hg if renal insufficiency or heart failure is present; or <130/80 mm Hg if diabetes is present.	AHA 2002 (1)	yes	
hypertension	TLC for pre-hypertension and hypertension	JNC7 2003 (2)	yes	
hypertension	Patients with a systolic blood pressure of 130–139 mm Hg or a diastolic blood pressure of 80–89 mm Hg may be given lifestyle therapy alone for a maximum of 3 months and then, if targets are not achieved, be treated with addition of pharmacological agents. (E)	ADA 2011 (4)	yes	
hypertension	Dietary Approaches to Stop Hypertension (DASH) diet (diet rich in fruits, vegetables, and low-fat dairy products, and reduced content of saturated and total fat)	JNC7 2003 (2)	yes	
hypertension	dietary sodium reduction (<=100 mmol per day)	JNC7 2003 (2)	yes	
hypertension	Dietary Approaches to Stop Hypertension (DASH)-style dietary pattern including reducing sodium and increasing potassium intake	ADA 2011 (4)	yes	
hypertension	physical activity (aerobic physical activity, e.g. brisk walking, 30 minutes per day for most days of the week)	JNC7 2003 (2)	yes	
hypertension	increased physical activity.	ADA 2011 (4)	yes	
hypertension	weight reduction (maintain BMI 19-25 kg/m^2)	JNC7 2003 (2), ADA 2011 (4)	yes	
hypertension	stop tobacco smoking	JNC7 2003 (2)	yes	
hypertension	moderation of alcohol intake (no more than 2 drinks (men) or 1 drink (women) per day	JNC7 2003 (2), ADA 2011 (4)	yes	
hypertension	For persons with renal insufficiency or heart failure, initiate drug therapy if BP is <130 mm Hg systolic or 85 mm Hg diastolic (<80 mm Hg diastolic for patients with diabetes). Initiate drug therapy for those with BP <140/90 mm Hg if 6 to 12 months of lifestyle modification is not effective, depending on the number of risk factors present.	AHA 2002 (1)	yes	
hypertension	Pharmacologic therapy for patients with diabetes and hypertension should be with a regimen that includes either an ACE inhibitor or an ARB. If one class is not tolerated, the other should be substituted. If needed to achieve blood pressure targets, a thiazide diuretic should be added to those with an estimated GFR (eGFR) >30 ml/min/1.73 m^2 and a loop diuretic for those with an eGFR <30 ml/min/1.73 m^2. (C)	ADA 2011 (4)	yes	ACEi or ARB as first-line treatment of elevated blood pressure in diabetes

Supporting information

guideline	Recommendations	Reference	Adopted by Siempre Salud	Note
hypertension	In pregnant patients with diabetes and chronic hypertension, blood pressure target goals of 110–129/65–79 mm Hg are suggested in the interest of long-term maternal health and minimizing impaired fetal growth. ACE inhibitors and ARBs are contraindicated during pregnancy. (E)	ADA 2011 (4)	yes	
hypertension	If blood pressure is >20/10 above goal, consider initiating treatment with two agents, one of which should be thiazide diuretic	JNC7 2003 (2)	yes	
hypertension	Thiazide diuretics initial therapy for most. Consider ACEi, ARB, BB, CCB as needed. Two-drug combination including a thiazide diuretic is initial treatment for stage 2 hypertension (BP >=160/100)	JNC7 2003 (2)	yes	
hypertension	See JNC7 2003, "Table 4. Oral Antihypertensive Drugs" and "Figure. Antihypertensive Treatment Algorithm" (2). Siempre Salud formulary to have one agent from each of four classes (thiazide diuretic, ACEi, dihydropyridine CCB, and BB).	JNC7 2003 (2)	yes	
hypertension	Patients with more severe hypertension (systolic blood pressure 140 or diastolic blood pressure 90 mmHg) at diagnosis or follow-up should receive pharmacologic therapy in addition to lifestyle therapy. (A)	ADA 2011 (4)	yes	
hypertension	Multiple drug therapy (two or more agents at maximal doses) is generally required to achieve blood pressure targets. (B)	ADA 2011 (4)	yes	
hypertension	Heart failure. Asymptomatic ventricular dysfunction: ASEi, BB. Symptomatic: ACEi, BB, ARB, aldosterone antagonist, loop diuretics	JNC7 2003 (2)	yes	
hypertension	Diabetes: Thiazide, BB, ACEi, ARB, CCB reduce CVD risk. ACEi/ARB decreases progression of diabetic nephropathy and reduce albuminuria. ARBs reduce progression to macroalbuminuria.	JNC7 2003 (2)	yes	
hypertension	Chronic kidney disease: ACEi/ARB reduce progression of nondiabetic renal disease. GFR <30 ml/min (Cr 2.5-3 mg/dl) requires increasing doses of loop diuretics in addition to other drug classes.	JNC7 2003 (2)	yes	
hypertension	Cerebrovascular disease: recurrent stroke risk reduced by combination of ACEi and thiazide diuretic.	JNC7 2003 (2)	yes	
hypertension	Thiazide diuretics should be used cautiously in patients who have gout or who have a history of significant hyponatremia.	JNC7 2003 (2)	yes	
hypertension	BBs should generally be avoided in individuals who have asthma, reactive airways disease, or second or third degree heart block.	JNC7 2003 (2)	yes	

Supporting information

guideline	Recommendations	Reference	Adopted by Siempre Salud	Note
hypertension	ACEIs and ARBs should not be given to women likely to become pregnant and are contraindicated in those who are pregnant. ACEIs should not be used in individuals with a history of angioedema.	JNC7 2003 (2)	yes	
hypertension	Aldosterone antagonists and potassium-sparing diuretics can cause hyperkalemia and should generally be avoided in patients who have serum potassium values more than 5.0 mEq/L while not taking medications.	JNC7 2003 (2)	yes	
hypertension	Resistant hypertension is the failure to reach BP goal despite adherence to full doses of an appropriate three-drug regimen that includes a diuretic. Explore identifiable causes of hypertension, consider consultation with subspecialist.	JNC7 2003 (2)	yes	
diabetes	Treat other risk factors more aggressively (e.g., BP goal <130/80 mm Hg)	AHA 2002 (1)	yes	
diabetes	Initiate appropriate hypoglycemic therapy to achieve near-normal fasting plasma glucose or as indicated by near-normal HbA1c. First step is diet and exercise. Second-step therapy is usually oral hypoglycemic drugs: sulfonylureas and/or metformin with ancillary use of acarbose and thiazolidinediones. Third-step therapy is insulin. Treat other risk factors more aggressively (eg, change BP goal to <130/80 mm Hg and LDL-C goal to <100 mg/dL).	AHA 2002 (1)	yes	Also see Siempre Salud standards of type 2 diabetes care
hyperlipidemia	Lifestyle change initiation: 10-year CVD risk >20%, initiate at LDL >=100; 10%-20%, initiate at LDL >=130; <10%, initiate at LDL >=160 mg/dl	ATP III 2002 (3)	yes	lab testing not available, initiate TLC in all patients with diabetes and/or hypertension
hyperlipidemia	Lifestyle modification (dietary) focusing on the reduction of saturated fat, *trans* fat, and cholesterol intake; increase of omega-3 fatty acids, viscous fiber, and plant stanols/sterols should be recommended to improve the lipid profile in patients with diabetes. (A)	ADA 2011 (4), ATP III 2002 (3)	yes	
hyperlipidemia	Increased physical activity	ADA 2011 (4), ATP III 2002 (3)	yes	
hyperlipidemia	Weight loss (if indicated)	ADA 2011 (4), ATP III 2002 (3)	yes	
hyperlipidemia	In patients with diabetes, statin therapy should be added to lifestyle therapy, regardless of baseline lipid levels: with overt CVD (A) and without CVD who are over age 40 years and have one or more other CVD risk factors, e.g. hypertension. (A)	ADA 2011 (4)	yes	Adopted but not implemented (statin cost-prohibitive)

Supporting information

guideline	Recommendations	Reference	Adopted by Siempre Salud	Note
hyperlipidemia	For patients with diabetes at lower risk than above (e.g., without overt CVD and under age 40 years), statin therapy should be considered in addition to lifestyle therapy if LDL cholesterol remains above 100 mg/dl or in those with multiple CVD risk factors. (E)	ADA 2011 (4)	yes	yes (multiple risk factor), no (LDL level). Adopted but not implemented (statin cost-prohibitive)
hyperlipidemia	Statin therapy is contraindicated in pregnancy. (E)	ADA 2011 (4)	yes	
primary prevention	CVD risk factors: smoking, hypertension, low HDL cholesterol (<40 mg/dl), family history premature CHD, age >=45 years (males) or >=55 years (females)	ATP III 2002 (3)	yes	
primary prevention	Goal: An overall healthy eating pattern.	AHA 2002 (1)	yes	
primary prevention	Goal: At least 30 min of moderate-intensity physical activity on most (and preferably all) days of the week.	AHA 2002 (1)	yes	
primary prevention	Goal: Complete cessation of tobacco smoking	AHA 2002 (1)	yes	
primary prevention	Goal: Achieve and maintain desirable weight (body mass index 18.5–24.9 kg/m^2).	AHA 2002 (1)	yes	
primary prevention	Advocate consumption of a variety of fruits, vegetables, grains, low-fat or nonfat dairy products, fish, legumes, poultry, and lean meats. Match energy intake with energy needs and make appropriate changes to achieve weight loss when indicated. Modify food choices to reduce saturated fats (<10% of calories), cholesterol (<300 mg/d), and trans-fatty acids by substituting grains and unsaturated fatty acids from fish, vegetables, legumes, and nuts. Limit salt intake to <6 g/d. Limit alcohol intake (<=2 drinks/d in men, <=1 drink/d in women) among those who drink.	AHA 2002 (1)	yes	
primary prevention	reduction of sodium intake	AHA 2002 (1)	yes	
primary prevention	consumption of fruits, vegetables, and low-fat dairy products;	AHA 2002 (1)	yes	
primary prevention	If cardiovascular, respiratory, metabolic, orthopedic, or neurological disorders are suspected, or if patient is middle-aged or older and is sedentary, consult physician before initiating vigorous exercise program. Moderate-intensity activities (40% to 60% of maximum capacity) are equivalent to a brisk walk (15–20 min per mile). Additional benefits are gained from vigorous-intensity activity (>60% of maximum capacity) for 20–40 min on 3–5 d/wk. Recommend resistance training with 8–10 different exercises, 1–2 sets per exercise, and 10–15 repetitions at moderate intensity >2 d/wk. Flexibility training and an increase in daily lifestyle activities should complement this regimen.	AHA 2002 (1)	yes	

Supporting information

guideline	Recommendations	Reference	Adopted by Siempre Salud	Note
primary prevention	When body mass index is >25 kg/m2, waist circumference at iliac crest level >40 inches in men, >35 inches in women. Initiate weight-management program through caloric restriction and increased caloric expenditure as appropriate. For overweight/obese persons, reduce body weight by 10% in first year of therapy.	AHA 2002 (1)	yes	
primary prevention	No exposure to environmental tobacco smoke. Ask about tobacco use status at every visit. In a clear, strong, and personalized manner, advise every tobacco user to quit. Assess the tobacco user’s willingness to quit. Assist by counseling and developing a plan for quitting. Arrange follow-up, referral to special programs, or pharmacotherapy. Urge avoidance of exposure to secondhand smoke at work or home.	AHA 2002 (1)	yes	initiate therapy with nicotine substitution, varenicline, or bupropion if available and affordable.
primary prevention	Consider aspirin therapy (75–162 mg/ day) as a primary prevention strategy in those with type 1 or type 2 diabetes at increased cardiovascular risk (10-year risk >=10%).	ADA 2011 (4)	yes	
primary prevention	Do not recommend for patients with aspirin intolerance. Low-dose aspirin increases risk for gastrointestinal bleeding and hemorrhagic stroke. Do not use in persons at increased risk for these diseases. Benefits of cardiovascular risk reduction outweigh these risks in most patients at higher coronary risk.Doses of 75–160 mg/d are as effective as higher doses. Therefore, consider 75–160 mg aspirin per day for persons at higher risk (especially those with 10-y risk of CHD of >=10%).	AHA 2002 (1)	yes	
primary prevention	Goal: Low-dose aspirin in persons at higher CHD risk (especially those with 10-y risk of CHD >=10%).	AHA 2002 (1)	yes	
primary prevention	Irregular pulse should be verified by an electrocardiogram. Conversion of appropriate individuals to normal sinus rhythm. For patients in chronic or intermittent atrial fibrillation, use warfarin anticoagulants to INR 2.0–3.0 (target 2.5). Aspirin (325 mg/d) can be used as an alternative in those with certain contraindications to oral anticoagulation. Patients <65 y of age without high risk may be treated with aspirin.	AHA 2002 (1)	yes	Aspirin (yes), warfarin (no). Lab testing not available.
hypertension	electrocardiogram	JNC7 2003 (2)	no	test not available
hypertension	hematocrit	JNC7 2003 (2)	no	test not available
hypertension	serum potassium	JNC7 2003 (2)	no	test not available
hypertension	serum creatinine	JNC7 2003 (2)	no	test not available
hypertension	calcium	JNC7 2003 (2)	no	test not available

Supporting information

guideline	Recommendations	Reference	Adopted by Siempre Salud	Note
hypertension	lipid profile	JNC7 2003 (2)	no	test not available
hypertension	urine albumin/creatinine ratio (optional)	JNC7 2003 (2)	no	test not available
hypertension	If ACE inhibitors, ARBs, or diuretics are used, kidney function and serum potassium levels should be monitored. (E)	ADA 2011 (4)	no	lab testing not available
hypertension	CVD risk >=20% to <30%, pharmacologic treatment to target <140/90; CVD risk >=30%, treatment to target <130/80	WHO 2007 (5)	no	use the AHA 2002, JNC7 2003, ADA 2011 more stringent criteria
hypertension	antihypertensives for anyone with bp >=160/100, and for CVD risk >=20% and bp >=140/90	WHO PEN 2010 (6)	no	use the AHA 2002, JNC7 2003, ADA 2011 more stringent criteria
diabetes	Treat other risk factors more aggressively (e.g., LDL-C goal <100 mg/dL).	AHA 2002 (1)	no	LDL testing not available
diabetes	Diabetes goals: Normal fasting plasma glucose (<110 mg/dL) and near normal HbA1c <7%).	AHA 2002 (1)	no	use less stringent ADA 2011 goal of fasting glucose <130 mg/dl.
hyperlipidemia	Screening In most adult patients, measure fasting lipid profile at least annually. In adults with low-risk lipid values (LDL cholesterol <100 mg/dl, HDL cholesterol >50 mg/dl, and triglycerides <150 mg/dl), lipid assessments may be repeated every 2 years. (E)	ADA 2011 (4)	no	lab testing not availalble
hyperlipidemia	Rule out secondary causes of hyperlipidemia (diabetes, hypothyroidism, obstructive liver disease, chronic renal failure, drugs (progestins, anabolic steroids, and corticosteroids)	ATP III 2002 (3)	no	laboratory evaluation not available
hyperlipidemia	If LDL-C is above goal range, rule out secondary causes (liver function test, thyroid-stimulating hormone level, urinalysis).	AHA 2002 (1)	no	
hyperlipidemia	CHD and CHD equivalents or Framingham Heart Study (FHS) 10-year CVD risk >20%, treatment goal LDL <100 mg/dl; Multiple (2+) risk factors or FHS CVD risk >=10% to <=20%, treatment goal LDL <130 mg/dl; 0-1 risk factor or FHS CVD risk <10%, treatment goal LDL <160 mg/dl.	ATP III 2002 (3)	no	lab testing unavailable
hyperlipidemia	LDL goals <130 if >=2 risk factors and CHD risk <20%; <100 if >=2 risk factors and CHD risk >=20%, respectively. LDL goal in diabetes is <100.	AHA 2002	no	lab testing unavailable

Supporting information

guideline	Recommendations	Reference	Adopted by Siempre Salud	Note
hyperlipidemia	Lipid goals: LDL-C <160 mg/dL if <=1 risk factor is present; LDL-C <130 mg/dL if >=2 risk factors are present and 10-y CHD risk is >20%; or LDL-C <100 mg/dL if >=2 risk factors are present and 10-y CHD risk is >20% or if patient has diabetes. Secondary goals (if LDL-C is at goal range): If triglycerides are <200 mg/dL, then use non-HDL-C as a secondary goal: non-HDL-C <190 mg/dL for <=1 risk factor; non-HDL-C <160 mg/dL for >=2 risk factors and 10-y CHD risk <20%; non-HDL-C <130 mg/dL for diabetics or for >=2 risk factors and 10-y CHD risk >20%. Other targets for therapy: triglycerides <150 mg/dL; HDL-C >40 mg/dL in men and >50 mg/dL in women.	AHA 2002 (1)	no	lab testing not available.
hyperlipidemia	In diabetic individuals without overt CVD, the primary goal is an LDL cholesterol <100 mg/dl (2.6 mmol/l). (A) In individuals with overt CVD, a lower LDL cholesterol goal of <70 mg/dl (1.8 mmol/l), using a high dose of a statin, is an option. (B) If drug-treated patients do not reach the above targets on maximal tolerated statin therapy, a reduction in LDL cholesterol of <30–40% from baseline is an alternative therapeutic goal. (A) Triglyceride levels <150 mg/dl (1.7 mmol/l) and HDL cholesterol >40 mg/dl (1.0 mmol/l) in men and >50 mg/dl (1.3 mmol/l) in women, are desirable. However, LDL cholesterol– targeted statin therapy remains the preferred strategy. (C)	ADA 2011 (4)	no	testing not available
hyperlipidemia	If LDL-C is above goal range, initiate additional therapeutic lifestyle changes consisting of dietary modifications to lower LDL-C: <7% of calories from saturated fat, cholesterol <200 mg/d, and, if further LDL-C lowering is required, dietary options (plant stanols/sterols not to exceed 2 g/d and/or increased viscous [soluble] fiber [10–25 g/d]),	AHA 2002 (1)	no	testing not available
hyperlipidemia	Lifestyle changes. Re-evaluated in six weeks. If goal not achieved, plants sterols/stanols, increased fiber, dietician referrral. Re-evaluate in six weeks. if goal not achieved (after total of 12 weeks), add medical therapy	ATP III 2002 (3)	no	lab testing not available for goal evaluation. Dietician not available.

Supporting information

guideline	Recommendations	Reference	Adopted by Siempre Salud	Note
hyperlipidemia	After 12 weeks of therapeutic lifestyle change, consider LDL-lowering drug therapy if: >=2 risk factors are present, 10-y risk is >20%, and LDL-C is >=130 mg/dL; >=2 risk factors are present, 10-y risk is <20%, and LDL-C is >=160 mg/dL; or <=1 risk factor is present and LDL-C is >=190 mg/dL. Start drugs and advance dose to bring LDL-C to goal range, usually a statin but also consider bile acid–binding resin or niacin. If LDL-C goal not achieved, consider combination therapy (statin-resin, statin-niacin). After LDL-C goal has been reached, consider triglyceride level: If 150–199 mg/dL, treat with therapeutic lifestyle changes. If 200–499 mg/dL, treat elevated non-HDL-C with therapeutic lifestyle changes and, if necessary, consider higher doses of statin or adding niacin or fibrate. If >500 mg/dL, treat with fibrate or niacin to reduce risk of pancreatitis. If HDL-C is <40 mg/dL in men and <50 mg/dL in women, initiate or intensify therapeutic lifestyle changes. For higher-risk patients, consider drugs that raise HDL-C (eg, niacin, fibrates, statins).	AHA 2002 (1)	no	lab testing and medications not available
hyperlipidemia	Treatment visits frequency: if LDL goal not met after TLC, start statin, bile acid sequestrant, or nicotinic acid. Re-evaluate in six weeks. If LDL goal not met, increase dose of statin or add bile sequestrant, or nicotinic acid. Re-evaluate in 6 weeks. if LDL goal not met, intensify therapy (add agent or increase dose) or refer to sub-specialist.	ATP III 2002 (3)	no	medication, lab testing not available
hyperlipidemia	Diabetes and CVD risk >=20%, give statin.	WHO PEN 2010 (6)	no	use the ADA 2011 criteria based on age and >=1 CVD risk factor
hyperlipidemia	In diabetes, if targets are not reached on maximally tolerated doses of statins, combination therapy using statins and other lipid lowering agents may be considered to achieve lipid targets but has not been evaluated in outcome studies for either CVD outcomes or safety. (E)	ADA 2011 (4)	no	testing not available
hyperlipidemia	10-year CVD risk >20%, initiate at LDL >=130; 10%-20%, initiate at LDL >=160; <10%, initiate at LDL >=190 mg/dl (initiate at >=160 mg/dl optional)	ATP III 2002 (3)	no	medication, lab testing not available
hyperlipidemia	HMG-CoA reductase inhibitors (statins), e.g. simvastatin (20-80 mg), atorvastatin 10-80 mg), etc., best effect on LDL	ATP III 2002 (3)	no	medications not available
hyperlipidemia	nicotinic acid, best effect on HDL (increased)	ATP III 2002 (3)	no	medications not available
hyperlipidemia	nicotinic acid and fibric acids, best effect on TG (decreased)	ATP III 2002 (3)	no	medications not available
hyperlipidemia	bile acid sequestrants, modest effect on LDL and HDL, no effect on TG	ATP III 2002 (3)	no	medications not available

Supporting information

guideline	Recommendations	Reference	Adopted by Siempre Salud	Note
hyperlipidemia	CVD risk >=30%, give statin. Target cholesterol <5.0 mmol/L (LDL <3.0 mmol/L) or lower cholesterol by 25% (LDL by 30%) whichever is greater	WHO 2007 (5)	no	medication, lab testing not available
hyperlipidemia	CVD risk 20% to <30%, age >40 with cholesterol >5.0 mmol/L (LDL >3.0 mmol/L), treat with a statin	WHO 2007 (5)	no	medication, lab testing not available
hyperlipidemia	non-diabetic patients, CVD risk >=30%, give statin (regardless of cholesterol or LDS levels)	WHO PEN 2010 (6)	no	statin not available
primary prevention	Diabetes and CVD risk >=20%, or non-diabetic and CVD risk >=30%, then low-dose aspirin	WHO PEN 2010 (6)	no	use the more stringent 10% risk cut-off of AHA 2002, ADA 2011
primary prevention	CVD risk >=30%, then low-dose aspirin	WHO 2007 (5)	no	use the more stringent 10% risk cut-off of AHA 2002, ADA 2011
primary prevention	Goals: Normal sinus rhythm or, if chronic atrial fibrillation is present, anticoagulation with INR 2.0–3.0 (target 2.5).	AHA 2002 (1)	no	warfarin and lab testing not available

References:

(1) Pearson TA, Blair SN, Daniels SR, Eckel RH, Fair JM, Fortmann SP, et al. AHA Guidelines for Primary Prevention of Cardiovascular Disease and Stroke: 2002 Update: Consensus Panel Guide to Comprehensive Risk Reduction for Adult Patients Without Coronary or Other Atherosclerotic Vascular Diseases. American Heart Association Science Advisory and Coordinating Committee. Circulation. 2002;106(3):388-91.

(2) Chobanian AV, Bakris GL, Black HR, Cushman WC, Green LA, Izzo JL, Jr., et al. The Seventh Report of the Joint National Committee on Prevention, Detection, Evaluation, and Treatment of High Blood Pressure: the JNC 7 report. JAMA. 2003;289(19):2560-72.

(3) Executive Summary of The Third Report of The National Cholesterol Education Program (NCEP) Expert Panel on Detection, Evaluation, And Treatment of High Blood Cholesterol In Adults (Adult Treatment Panel III). JAMA. 2001;285(19):2486-97.

(4) American Diabetes Association. Standards of Medical Care in Diabetes—2011. Diabetes Care. 2011;34(Supplement 1):S11-S61.

(5) World Health Organization. Prevention of cardiovascular disease. Guidelines for the assessment and management of total cardiovascular risk. Geneva, Switzerland: World Health Organization; 2007.

(6) World Health Orgnization. Package of Essential Noncommunicable (PEN) Disease Interventions for Primary Health Care in Low-Resource Settings. Geneva, Switzerland: World Health Organization; 2010. 1-66 p.
