# Supplementary material for: A retrospective cohort study of a community-based primary care program’s effects on pharmacotherapy quality in low-income Peruvians with type 2 diabetes and hypertension
Source: PLOS Glob Public Health. 2024 Aug 22;4(8):e0003512. doi: 10.1371/journal.pgph.0003512 (PMC11341050; doi:10.1371/journal.pgph.0003512)
Supplement: S2 Table — (PDF) [file pgph.0003512.s005.pdf]

Supporting Information

S2 Table. Siempre Salud medication treatment profiles for type 2 diabetes and hypertension

Stage 1 hypertension, serum potassium available

Co-morbidity	Baseline	Target
None †, serum potaassium available	SBP 140-159 and DBP 90-99 mm Hg	SBP<140 and DBP <90 mm Hg

Drug	Dose	Maximum daily dose		Time (weeks) ¶
First-line thiazide diuretic			†	
HCTZ	25 mg qD	50 mg	†	
	↓		†	2 to 4
Add ARB (or ACEi) *			†	
Losartan	50 mg qD	100 mg	†	
	↓		†	2 to 4
Increase thiazide			†	
HCTZ	50 mg qD	50 mg	†	
	↓		†	2 to 4
Increase ARB (or ACEi)			†	
Losartan	100 mg qD	100 mg	†	
	↓			2 to 4
Add calcium-channel blocker ‡‡				
Amlodipine	5 mg qD			
	↓			2 to 4
Increase calcium-channel blocker ‡‡				
Amlodipine	10 mg qD	10 mg		
	↓			2 to 4
Add beta-adrenergic blocker				
Atenolol	50 mg qD			
	↓			2 to 4
Atenolol	100 mg qD	100 mg		
	↓			
refer to subspecialist				14 to 28 total weeks

Stage 1 hypertension, serum potassium not available

Co-morbidity	Baseline	Target
None †, serum potaassium unavailable	SBP 140-159 and DBP 90-99 mm Hg	SBP<140 and DBP <90 mm Hg

Drug	Dose	Maximum daily dose		Time (weeks) ¶
First-line calcium-channel blocker ‡‡				
Amlodipine	5 mg qD			
	↓			2 to 4
Increase calcium-channel blocker ‡‡				
Amlodipine	10 mg qD	10 mg		
	↓			2 to 4
Add thiazide diuretic			†	
HCTZ	25 mg qD	50 mg	†	
	↓		†	2 to 4
Add ARB (or ACEi) *			†	
Losartan	50 mg qD	100 mg	†	
	↓		†	2 to 4
Increase thiazide			†	
HCTZ	50 mg qD	50 mg	†	
	↓		†	2 to 4
Increase ARB (or ACEi)			†	
Losartan	100 mg qD	100 mg	†	
	↓			2 to 4
Add beta-adrenergic blocker				
Atenolol	50 mg qD			
	↓			2 to 4
Atenolol	100 mg qD	100 mg		
	↓			
refer to subspecialist				14 to 28 total weeks

ACEi = angiotensin converting enzyme inhibitor, HCTZ = hydrochlorthiazide, ARB = angiotension receptor blocker

* if using ACEi, e.g., enalapril, then start with 10 mg qD and increase by 10 mg mg every 2-4 weeks to 20 mg BID (max daily dose 40 mg)

‡ ARB or ACEi recommended as first-line treatment in diabetes (ADA). ARB or ACEi delays onset of microalbumnuria (1).

§ initial combination therapy recommended for stage 2 hypertension (2)

¶ AHA/ACC and WHO both recommend monthly follow-up until BP controlled

‡‡ CCBs instead of ACEi/ARB or thiazide diuretics as first-line treatment when laboratorty testing for serum potassium unavailable

References

1 American Diabetes Association. Standards of Medical Care in Diabetes—2011. Diabetes Care. 2011;34(Supplement 1):S11-S61. doi: 10.2337/DC11-S011.

2 Gradman AH, Basile JN, Carter BL, Bakris GL. Combination Therapy in Hypertension. The Journal of Clinical Hypertension. 2011;13(3):146-54. doi: 10.1111/j.1751-7176.2010.00397.x.

Supporting Information

S2 Table. Siempre Salud medication treatment profiles for type 2 diabetes and hypertension

Stage 1 hypertension, type 2 diabetes

Co-morbidity	Baseline	Target
Diabetes with normal GFR ‡	SBP 130-159 and DBP 80-99 mm Hg	SBP<130 and DBP <80 mm Hg

Stage 2 hypertension +/- diabetes

Co-morbidity	Baseline	Target
None, or diabetes with normal GFR	SBP >=160 or DBP >=100 mm Hg (>=150/90 in diabetes) §	SBP<140 and DBP <90 mm Hg (<130/80 in diabetes)

Drug	Dose	Maximum daily dose	Time (weeks) ¶
First-line ARB (or ACEi) * ‡			
Losartan	50 mg qD	100 mg	
	↓		2 to 4
Increase ARB (or ACEi) * ‡			
Losartan	100 mg qD	100 mg	
	↓		2 to 4
Add thiazide diuretic ††			
HCTZ	25 mg qD	50 mg	
	↓		2 to 4
Increase thiazide ††			
HCTZ	50 mg qD	50 mg	
	↓		2 to 4
Add calcium-channel blocker			
Amlodipine	5 mg qD		
	↓		2 to 4
Increase calcium-channel blocker			
Amlodipine	10 mg qD	10 mg	
	↓		2 to 4
Add beta-adrenergic blocker			
Atenolol	50 mg qD		
	↓		2 to 4
Atenolol	100 mg qD	100 mg	
	↓		
refer to subspecialist			14 to 28 total weeks

Drug	Dose	Maximum daily dose	Drug	Dose	Maximum daily dose	Time (weeks) ¶
First-line dual thiazide diuretic and ARB						
HCTZ	25 mg qD	50 mg	Losartan	50 mg qD	100 mg	
	↓			↓		2 to 4
Increase ARB						
			Losartan	100 mg qD	100 mg	
				↓		2 to 4
Increase thiazide diuretic						
HCTZ	50 mg qD	50 mg				
	↓					2 to 4
Add calcium-channel blocker						
Amlodipine	5 mg qD					
	↓					2 to 4
Increase calcium-channel blocker						
Amlodipine	10 mg qD	10 mg				
	↓					2 to 4
Add beta-adrenergic blocker						
Atenolol	50 mg qD					
	↓					2 to 4
Atenolol	100 mg qD	100 mg				
	↓					
refer to subspecialist						12 to 24 total weeks

Supporting Information

S2 Table. Siempre Salud medication treatment profiles for type 2 diabetes and hypertension

Stage 1 hypertension, ischemic heart disease

Co-morbidity	Baseline	Target
Ischemic heart disease	SBP 140-159 and DBP 90-99 mm Hg	SBP<140 and DBP <90 mm Hg

Drug	Dose	Maximum daily dose	Drug	Dose	Maxium daily dose	Time (weeks) ¶
First-line ACEi (or ARB *) + beta-blocker + aspirin + statin						
Losartan	50 mg qD		Atenolol	50 mg qD		
	↓			↓		2 to 4
Losartan	100 mg qD	100 mg	Atenolol	100 qD	100 mg	
Add thiazide diuretic						
HCTZ	25 mg qD					
	↓		↓			2 to 4
HCTZ	50 mg qD	50 mg				
	↓		↓			2 to 4
Add calcium-channel blocker						
Amlodipine	5 mg qD					
	↓		↓			2 to 4
Amlodipine	10 mg qD	10 mg				
	↓		↓			2 to 4
refer to subspecialist						10 to 20 total weeks

Stage 1 hypertension, cerebrovascular disease

Co-morbidity	Baseline	Target
Cerebrovascular disease	SBP 140-159 and DBP 90-99 mm Hg	SBP<140 and DBP <90 mm Hg

Drug	Dose	Maximum daily dose	Time (weeks) ¶
First-line thiazide diuretic + aspirin + statin			
HCTZ	25 mg qD		
	↓		2 to 4
HCTZ	50 mg qD	50 mg	
	↓		2 to 4
Add ACEi (or ARB *)			
Losartan	50 mg qD		
	↓		2 to 4
Losartan	100 mg qD		
	↓		2 to 4
Add calcium-channel blocker			
Amlodipine	5 mg qD		
	↓		2 to 4
Amlodipine	10 mg qD	10 mg	
	↓		2 to 4
Add beta-adrenergic blocker			
Atenolol	50 mg qD		
	↓		2 to 4
Atenolol	100 mg qD	100 mg	
	↓		
refer to subspecialist			14 to 28 total weeks

Treatment with hypoglycemic agents

Medication	Dose	Alternative dose	Maximum daily dose	Time (weeks)
First-line agent (metformin)				
Metformin	500 mg qD	850 mg qD		
	↓	↓		2 to 4
Metformin	500 mg BID	850 mg BID		
	↓	↓		2 to 4
Metformin	1000 mg BID	850 mg TID		
	↓	↓		2 to 4
Metformin	850 mg TID	↓	2550 mg	
	↓	↓		2 to 4
Failed monotherapy				
Begin sulfonylurea †				
Glibenclamide ‡	5 mg qD			
	↓			2 to 4
Glibenclamide ‡	5 mg BID			
	↓			2 to 4
Glibenclamide ‡	5 mg TID			
	↓			2 to 4
Glibenclamide ‡	10 mg BID		20 mg	
	↓			
Failed dual therapy				14 28 total (weeks)
Begin triple therapy at 6 months. If resources available, add thiazolidinedione, DPP-4 inhibitor, SGLT2 inhibitor, or injectables, GLP-1 receptor agonist or basal insulin (glargine or detemir).				

† ADA recommends adding 2nd medication if metformin monotherapy has not achieved control in 3 months

‡ Glibenclamide (long-acting, renal elimination) is listed because of its availability but glicazide (intermediate-acting, hepatic elimination) is preferred because of its better safety profile, especially in older patients and those with renal insufficiency
